# Extracellular Vesicles’ Role in the Pathophysiology and as Biomarkers in Cystic Fibrosis and COPD

**DOI:** 10.3390/ijms24010228

**Published:** 2022-12-23

**Authors:** Sante Di Gioia, Valeria Daniello, Massimo Conese

**Affiliations:** Department of Clinical and Experimental Medicine, University of Foggia, 71122 Foggia, Italy

**Keywords:** extracellular vesicles, apoptotic bodies, microvesicles, exosomes, miRNAs, cystic fibrosis, chronic obstructive pulmonary disease, pathomechanisms

## Abstract

In keeping with the extraordinary interest and advancement of extracellular vesicles (EVs) in pathogenesis and diagnosis fields, we herein present an update to the knowledge about their role in cystic fibrosis (CF) and chronic obstructive pulmonary disease (COPD). Although CF and COPD stem from a different origin, one genetic and the other acquired, they share a similar pathophysiology, being the CF transmembrane conductance regulator (CFTR) protein implied in both disorders. Various subsets of EVs, comprised mainly of microvesicles (MVs) and exosomes (EXOs), are secreted by various cell types that are either resident or attracted in the airways during the onset and progression of CF and COPD lung disease, representing a vehicle for metabolites, proteins and RNAs (especially microRNAs), that in turn lead to events as such neutrophil influx, the overwhelming of proteases (elastase, metalloproteases), oxidative stress, myofibroblast activation and collagen deposition. Eventually, all of these pathomechanisms lead to chronic inflammation, mucus overproduction, remodeling of the airways, and fibrosis, thus operating a complex interplay among cells and tissues. The detection of MVs and EXOs in blood and biological fluids coming from the airways (bronchoalveolar lavage fluid and sputum) allows the consideration of EVs and their cargoes as promising biomarkers for CF and COPD, although clinical expectations have yet to be fulfilled.

## 1. Introduction

### 1.1. General Role of EVs in Various Pathological Processes

Cells secrete or shed small vesicles from their membrane called extracellular vesicles (EVs), functioning as a long distance cell-to-cell communication mechanism [1]. EVs mediate the transport of several molecules that can exert positive or detrimental effects on their target cells. Indeed, via both autocrine and paracrine signaling, EVs can induce many changes in the biological activity of cells, such as the activation of the inflammatory process, the increase in cell proliferation, etc. In recent years, the role of EVs in several pathological conditions has been clarified, and it is well known that their specific cargo is associated with different pathophysiological statuses. Hereafter, we give a brief overview of EVs’ roles in some pathological conditions. In the cardiovascular system, EVs can be secreted by different cell types (e.g., cardiomyocytes, smooth muscle cells, macrophages, monocytes, fibroblasts, and endothelial cells), and can trigger several pathological processes [2]. It must be said that the *primum movens* in the pathogenesis of many cardiovascular diseases is represented by the endothelial dysfunction associated with atherosclerosis. EVs secreted by various cells, such as leukocytes, platelets, smooth muscle cells and endothelial cells, can activate the endothelium, promoting monocyte adhesion and transmigration, all of which are fundamental processes in inducing atherosclerosis [3]. In particular, platelet-derived EVs are involved in several physiological processes such as endothelial cell proliferation, survival, migration, and vessel formation. Platelet EVs promote inflammation via their cargo, which contain mediators capable of activating the endothelium and facilitating cell–cell interaction. Some studies have demonstrated that platelet-derived EVs are able of promoting tissue repair after cardiac damage [4].

Liver is another organ in which the pathophysiological role of EVs has been clarified. EVs secreted by hepatocytes, Kupffer cells, cholangiocytes, hepatic stellate cells and liver sinusoidal endothelial cells, and they exert an important role in many process such as liver injury, regeneration, fibrosis, inflammation, ductular reaction, and cancer development [5].

Emerging studies suggest a role for EVs both in neuroinflammation and neurodegeneration. EVs have both beneficial and detrimental roles in neuroinflammation [3]. Indeed, EVs act as “shuttles” of pro-inflammatory mediators, facilitating their transport from damaged cells to neighboring naïve neural cells [6]. Microglial cells, which are the first to respond during neuroinflammation, once activated can proliferate and in turn release EVs, allowing the spreading of danger signals across the brain [7]. In neurodegenerative diseases, such as Alzheimer’s disease (AD) and Parkinson’s diseases (PD), EVs work as carriers of pathogenic proteins. Indeed, in the case of AD and PD, EVs allow the spreading of the causative agents: αβ peptide and phosphorylated tau and α-Syn, respectively.

In cancer research, many studies have shown that EVs contribute to tumor progression by promoting metastatic dissemination and resistance to chemotherapy [8]. EVs from tumor cells are involved in the crosstalk between stromal cells and cancer cells as well as in reprogramming cancer cells, promoting their epithelial–mesenchymal transition. Moreover, EVs can encapsulate microRNAs (miRNAs) and proteins, which can transform nonmalignant cells or improve chemotherapy resistance. EVs can also alter the expression of molecules involved in the immune response against cancer cells. All of these studies demonstrated that EVs can “educate” cancer cells by means of their cargo, in particular proteins and miRNAs.

Finally, EVs have an important role in infectious diseases where the crosstalk between host cells and pathogenic organisms is fundamental [8]. In vitro, it has been demonstrated that EVs, derived from hepatoma cells infected with HCV, promote infection without interaction between target cells and viruses. Moreover, there is an increasing interest in studying EVs as a marker of infection. Interestingly, studies concerning HIV-1-infected patients have demonstrated that the quantity of EVs is informative of the activity of viral infection. Again, the modification of the EVs’ cargo, in terms of microRNAs, can be a molecular signature of response to therapy.

In respiratory medicine, a large body of studies has demonstrated the involvement of EVs in the pathogenesis of lung diseases such as lung cancer, pulmonary fibrosis and SARS-CoV-2 infection. Indeed, EVs’ cargo can vary in terms of the dependence of the different pathophysiological status of the disease [9]. In lung cancer, EVs secreted by malignant cells have important roles in oncogenesis and tumor progression [10]. Lung cancer cells’ EVs are involved in the stimulation of angiogenesis, enhancing the expression of vascular endothelial growth factor (VEGF) [11]. Moreover, cancer cells’ EVs can modify the “immunological microenvironment” of the tumor. Indeed, EVs can induce mesenchymal stem cells (MSCs) to acquire a pro-inflammatory phenotype, or induce a tolerogenic response to attenuate the killing activity of CD8 T cells, both of which are fundamental processes in tumor growth [12,13]. In addition, cancer cells’ EVs can promote the epithelial-to-mesenchymal transition [14] as well as the pre-metastatic niche [15]. In idiopathic pulmonary fibrosis (IPF), EVs have shown potential as biomarkers [9]. In particular, using a murine model of lung fibrosis, the role of an EV–miRNA as a potential marker of different stages of IPF was clarified [16]. Interestingly, the same miRNA was increased in the serum of IPF patients. As compared to conventional biomarkers of IPF, such as Krebs von den Lungen-6 (KL-6) and matrix metalloproteases, EVs and miRNAs seem to be quite specific to cell conditions in pulmonary fibrosis [9]. Recently, many studies were carried out to evaluate the role of EVs in SARS-CoV-2 infection [17]. It was demonstrated that several cytotypes represent the first source of EVs [18,19,20]. Interestingly, the presence of ACE2 (receptor angiotensin-converting enzyme 2) in EVs released by some types of cells [21] suggests an important role of these vesicles in promoting infection, given that ACE2 is fundamental for the fusion between SARS-CoV-2 viral particles and the host cell membrane. During respiratory infections by coronaviruses, the serum level of EVs increases [22,23]. Moreover, EVs may also activate host immune responses if they carry viral and self-antigens [24]. The systemic increase in exosomes reported and detected during SARS-CoV-2 infection [25,26] may be closely related to the pathological events characteristic of COVID-19. Indeed, it is well known that SARS-CoV-2 infection is associated with an increased risk of thromboembolic events. In COVID-19 patients, the contribution of platelet EVs to thrombotic events is mainly due to their higher expression of procoagulant proteins, such as tissue factor [4].

### 1.2. CF and COPD

EVs also contribute to the pathogenesis of chronic inflammatory respiratory diseases, such as asthma, cystic fibrosis (CF) and chronic obstructive pulmonary disease (COPD) [27]. CF and COPD share a lung pathology whose hallmarks are mucus overproduction, opportunistic bacterial infections and a chronic inflammatory response, eventually leading to bronchiectasis and lung failure. While CF is caused by mutations in the CF transmembrane conductance regulator gene (CFTR), COPD is considered an acquired CFTR dysfunction, mostly due to cigarette smoking. Indeed, these two diseases display a substantial overlap in clinical phenotype, and also more recently in the role of EVs in their pathophysiological processes. The aim of this review is to focus on the evidence of EVs’ role in the pathomechanisms of CF and COPD, as a novel portrait of these diseases that could also advance biomarker options.

#### 1.2.1. CF

CF is an autosomal recessive condition that affects various organs and tissues, and is the lung disease responsible for the majority of morbidity and mortality cases. The CFTR protein regulates the exchange of chloride, bicarbonate and sodium ions through epithelial membranes. More than 2000 variations have been recognized in the CFTR gene, among which only a limited subset is known to be disease-driven [28]. CFTR mutations have been classified into six classes, depending on the effect on CFTR protein maturation and transport to the plasma membrane, channel opening, gating and recycling [29]. The most frequent mutation, affecting ~80% of the Caucasian population, where CF shows the highest incidence, is the deletion of phenylalanine at position 508, i.e., *F508del*, causing misfolding, an altered interaction with chaperonins at the endoplasmic reticulum level, and premature proteasome-mediated degradation [30,31]. However, this class II mutation also causes defects belonging to class III (reduced channel gating) [32,33] and class VI (altered recycling) [34,35,36] mutations.

In CF, CFTR loss/malfunction causes a disrupted ion and fluid flux through the airway epithelium resulting in the production of thick/viscous mucus, which is due to the pathological increment in proteins, mucin and biological polymers [37]. Increased airway mucus viscosity, resulting in the mucopurulent obstruction of small and medium-size bronchioles and bronchiectasis [38], promotes bacterial infection and inflammation, which may proceed until patients die of respiratory insufficiency [39]. A neutrophil-dominated peribronchial and endobronchial inflammation is the hallmark of CF lung inflammatory disease, although the propagation of inflammation and damage to the lungs is not well known [40]. Although neutrophils are considered the major immune cell type responsible for inflammation and tissue damage in CF lungs [41], other innate immune cells have been found to be dysregulated in CF, such as epithelial cells, macrophages, and dendritic cells [42,43,44]. However, the interplay among these cell types is scarcely known. Nowadays, the clinical history of CF has been profoundly changed by the introduction to the therapeutic toolbox of small-molecule drugs modifying the expression and function of mutated CFTRs. To keep with the *F508del* mutation and its multiple alterations, a cocktail of correctors (inciting correct trafficking to the plasma membrane) and potentiators (increasing channel-opening probability) are assumed to control CF disease in all patients bearing at least *F508del* on at least one allele, representing nearly 90% of CF individuals [45].

#### 1.2.2. COPD

COPD is characterized by a spectrum of conditions, including small-airway inflammation with subsequent narrowing and emphysema, which causes irreversible damage to distal parts of the lung [46,47]. Cigarette smoke (CS) is the main cause of these pathologic alterations, resulting in inflammation and remodeling through the modification of bronchial epithelial cells, the differentiation of fibroblasts into myofibroblasts [48], and eventually the EVs produced by these cells [49]. Inflammation, oxidative stress, and protease–antiprotease disproportion are the hallmarks of the pathogenesis of chronic bronchitis, small-airway disease and emphysema [50].

COPD and CF have in common many clinical and pathologic features, such as the airflow limitation, neutrophilic inflammation and the enhancement of lung mucus viscosity [51,52]. Of the two classic COPD phenotypes, prevalent emphysema and prevalent chronic bronchitis (CB) [53,54], the latter exhibits pathologic features similar to CF, including mucin overproduction and mucus accumulation. The CB phenotype affects nearly two-thirds of COPD patients [55]. While CF is a genetic disease due to CFTR mutations, COPD is typically the result of smoke and the inhalation of toxic substances. A deficiency of α1-antitrypsin (α1AT), as a cause of COPD, is generally considered to be rare, and less than 3% of people with COPD have an α1AT deficiency [48]. Individuals with an α1AT deficiency are at a high risk of COPD, especially if they are smokers, due to unopposed elastase activation [56,57], as well as excessive apoptosis [58,59] and lung inflammation [60,61,62].

Recent in vitro and in vivo findings have determined that COPD is considered an obstructive lung disease due to acquired deficiency of CFTR. Mechanistically, CS-induced oxidative stress and increased cytosolic Ca^2+^ levels as well as CS toxic components, which are considered the major causes of COPD, can lead to reduced CFTR levels in airway epithelia [63]. CFTR loss/dysfunction is due to multiple mechanisms, including reduced CFTR transcription, diminished CFTR protein by heightened protein degradation, and an increase in its internalization rate followed by the retrograde trafficking of CFTR to the endoplasmic reticulum [64,65,66,67]. Among the toxic CS constituents, acrolein, cadmium and manganese are thought to cause CFTR dysfunctions in vitro and in vivo [68,69,70]. Moreover, CS could also alter CFTR lipid rafts in macrophages, impairing bacterial phagocytosis and killing activity [71]. Overall, these findings suggest that, as in CF, the first steps in COPD pathogenesis (especially regarding CB) are represented by impaired ion and liquid homeostasis, mucociliary clearance reduction, and the ensuing opportunistic infection and inflammation [63]. However, the COPD pathogenesis of early as well of chronic disease is not well known, leaving room for other pathomechanisms to be found and studied. The similarity of COPD to CF extends so far that CFTR modulators are being evaluated for CFTR rescue and clinical efficacy in COPD patients [72,73,74].

## 2. Extracellular Vesicles (EVs) and Lung Diseases

### 2.1. Biogenesis of EVs

Most prokaryotes and eukaryotic cells release EVs into the extracellular environment [75,76,77]. EVs consist of a phospholipid bilayer and have a role in cell-to-cell communication and disease pathogenesis [78,79]. As reported in the Minimal Information for Studies of Extracellular Vesicles (MISEV) guidelines, published by the International Society for Extracellular Vesicles (ISEV), depending on their size, density, composition (surface markers), biogenesis and secretory mechanisms [80], EVs can be distinguished into (1) apoptotic bodies, (2) microvesicles and (3) exosomes. Among these three structures, the apoptotic bodies are those with the largest size, in a range between 1000 and 5000 nm. Apoptotic bodies (ABs) derive from the fragmentation of the cell in the process of apoptosis and are released by budding from the plasma membrane. Apoptosis-derived EVs (ApoBDs) may contain cellular organelles, membranes, and cytosolic contents, but also noncoding RNAs and DNA fragments [79]. The density of ApoBDs is in the range between 1.18 and 1.28 g/mL [81]. Microvesicles (MVs), also known as microparticles (MPs) or ectosomes, are smaller than apoptotic bodies, ranging from 150–1000 nm. MVs originate from the outward budding of the plasma membrane and are subsequently released extracellularly [82]. These EVs are enriched with lipids, particularly phosphatidylserine (PS) [83,84]. The sucrose gradient density of MVs, lower than ApoBDs, is between 1.25 and 1.30 g/mL [85,86,87]. Exosomes (EXOs) are characterized by a double phospholipid layer and a diameter between 30 and 150 nm. The biogenesis of exosomes is a complex process that depends on mother-cell-stimulating signals [88] and begins with the formation of early endosomes [89,90,91]. The early sorting endosomes become late sorting endosomes [92] with the formation of intraluminal vesicles (ILVs) within the lumen of the endosome. Multivesicular bodies (MVBs) are formed thanks to ILVs which, during their development, randomly incorporate portions of the cytosol, as well as transmembrane and peripheral proteins, into the invaginating membrane [87]. Some MVBs are degraded in lysosomes by the endosomal sorting complex required for transports (ESCRTs)-dependent mechanism, while a good portion of these MVBs fuses with the plasma membrane and secretes exosomes via exocytosis into the extracellular space, using RAB GTPases (ESCRT-independent mechanism) [93,94].

The ESCRT mechanism, which is composed of the various ESCRT complexes (ESCRT-0, -I, -II, -III) and some proteins such as vacuolar protein sorting-associated protein 4 (Vps4), apoptosis-linked gene 2-interacting protein X (ALIX) and tumor susceptibility gene 101 protein (TSG101) [90,95], takes part in the process of the biogenesis/degradation of MVBs in a ubiquitination-dependent manner [96]. The ESCRT-0 complex is recruited to the endosomal membrane, while ESCRT-I and -II are required to cause the deformation of the membrane, allowing the sorting of cargo molecules in the MVBs. Subsequently, the ESCRT-III/Vps4 complex regulates the concentration of the components of the MVBs’ cargo and separates the vesicles from the plasma membrane [97,98].

Although the three main classes of EVs have been identified, as previously mentioned, as EXOs, MVs and ApoBDs, the issue of EV diversity has not been completely elucidated and is the subject of intense investigation by ISEV and independent researchers. Currently, it can be said that cells release a spectrum of heterogeneous populations of EVs with overlapping sizes [99]. Simply, it has been observed that EV subtypes can be differentiated according to: (1) physical characteristics, such as size or density; (2) biochemical composition; and (3) descriptions of conditions or cell of origin [100,101]. In terms of size, we can define small EVs (S-EVs) as being <200 nm in diameter and large and/or medium EVs (m/l-EVs or L-EVs) as over 200 nm [80]. As for the molecular content, these EVs differ since L-EVs have more DNA, CD9, and Annexin A1, while S-EVs carry CD63 and CD81 [100]. Moreover, it has been proposed that, concerning miRNAs, there exist different EV subpopulations with unique characteristics and miRNA contents [99]. Finally, other EV subpopulations, called large oncosomes and originating directly from the plasma membrane, are released specifically by cancer cells, and they are larger than any other EV, i.e., between 1 and 10 μm [102].

Besides the inherent heterogeneity of EVs, complexity also concerns EXOs, which can include different subpopulations based on their size, content (cargo), functional impact on recipient cells, and cell of origin [103]. Three types of EXOs have been identified according to their size (40–75 nm, 75–100 nm, or 100–160 nm), their biomarker (tetraspanins CD63, CD9, or CD81) and their functional heterogeneity. In this last case, one EXO subtype can induce cell survival, while another set induces apoptosis, and a different set induces immunomodulation, etc., in different target cell types [103,104]. To illustrate this complex diversity, Zhang et al. [105], by using asymmetric flow field-flow fractionation, identified two exosome subpopulations (large exosome vesicles, Exo-L, 90–120 nm; small exosome vesicles, Exo-S, 60–80 nm) and discovered an abundant population of nonmembranous nanoparticles termed “exomeres” (~35 nm), and each of these subpopulations were associated with specific phenotypes and cargoes. The heterogeneity in terms of both the size and composition of EXOs is not surprising given the different protein complexes involved in ESCRT machinery and the existence of the ESCRT-independent pathway of EXO biogenesis.

The assessment of EV heterogeneity is the focus of several methods that capture EV populations and subpopulations, including ultrafiltration, differential ultracentrifugation, gradient ultracentrifugation, precipitation, size-exclusion chromatography, immune-affinity capture, mass spectrometric immunoassay, etc. [80,99,106,107,108]. An initial characterization of EV subpopulations, obtained after isolation, can be performed morphologically by scanning electron microscopy (SEM) [109], transmission electron microscopy (TEM) [110,111,112] or atomic force microscopy (AFM) [77,80,106,108].

Since EXOs are the EV subpopulation characterized by a distinct intracellular regulatory process that likely affects their composition, and possibly their function, when secreted into the extracellular space and interacting with recipient cells [113,114,115], this review is intended to place particular emphasis on EXOs and to discuss other EVs when a thoughtful comparison is useful.

### 2.2. EVs’ Cargo

The Bradford or micro-bicinchoninic acid (BCA) protein assay is used to quantify the vesicular proteins that are subsequently separated and specifically detected by Western blot analysis using different monoclonal antibodies, i.e., CD9, CD63, CD81, Hsp70 [109], MHC II, CD40, CD80, CD86, and CD54 [77,80,106,108].

Purified EVs can thus undergo characterization with respect to their cargoes, such as metabolites and lipids [116,117,118], proteins [117,119], transcripts (prevalently mRNAs, miRNAs and long noncoding (lnc) RNAs, but also small nuclear RNA, small nucleolar RNA, noncoding RNA, long intergenic noncoding RNA, piwi-interacting RNA) [117,120,121], and genetic material (single-strand DNA, double-strand DNA, mitochondrial DNA) [117,122]. The vesicles’ cargo depends on the source from which the EVs originate and on their biological state (e.g., transformed, differentiated, stimulated, and stressed) [89,123]. Furthermore, based on the molecular content of EVs, it is possible to understand the physiological and pathological state of the cells [124,125,126].

Exosomes consist of a lipophilic shell with lipids and intramembrane ligands and receptors [127]. These vesicles are detected in biological fluids such as blood [128], sputum [129], cerebrospinal fluid [130], bronchial alveolar lavage fluid (BALF) [131], and urine [132], but they can also be secreted by various cell types such as bronchial epithelial cells [133], mesenchymal stem cells [134], macrophages [135], dendritic cells [136], natural killer (NK) cells [137], T cells [138], and B cells [139]. Exosomes are released into the extracellular environment and directed to different organs such as the lung, liver, kidney, pancreas, spleen, gastrointestinal tract, etc. [140,141,142]. Thanks to EXOs’ amphiphilic properties, hydrophobic and hydrophilic bioactive molecules can be incorporated into them [143].

EXOs have a very complex composition. They are characterized by a high and specific protein content (~4563 proteins) [144,145] derived from the plasma membrane, the cytosol, and the endocytosis pathway. Particularly, EXOs are rich in the most common proteins CD9, CD81, CD82, CD63, CD106 of the tetraspanin family [89,103,146], which act as specific membrane markers [147] and play an important role in cell penetration, invasion and fusion; the heat shock proteins HSP60, HSP70, HSP90, which allow the peptide to be loaded onto MHC I; Alix, Flotillin-1 and TSG101 proteins [89,103,146], which are necessary during the biogenesis of exosomes and useful for the trafficking and release through the MVB formation [103]. Among the various proteins that are part of the exosomes’ cargo there are those located on the surface of the vesicles that can induce intracellular signaling by interacting with the target cells’ receptors. Furthermore, EXOs carry other proteins such as annexins (I, II, V e VI) and Ras proteins that promote the docking of vesicles and membrane transport and fusion [145], cell-specific class II proteins of the myosin heavy chain, which are incorporated only in exosomes isolated from APC cells [148], GTPases (EEF1A1, eEF2) and cytoskeletal proteins (actin, sinenin and moesin), enzymes and signal transduction proteins as well as lipids (approximately 194) [103,145,149], and functionally active genetic material including DNA, messenger RNA (mRNA), microRNAs (miRNAs), noncoding RNA (tRNAs, rRNAs), and metabolites [150,151].

miRNAs are small noncoding RNAs ranging in size from 20 to 22 nucleotides and are the most abundant component of the exosome’s cargo. Their internalization in exosomes is regulated by the presence of RNA-binding proteins via specific conserved miRNA motifs. For example, there are various RNA-binding proteins, such as SUMO protein (hnRNBA1), that can recognize GAGAG motifs of miRNA [152], or the synaptotagmin-binding cytoplasmic RNA-interacting protein that identifies the GGCU motif [153]. Previously, several studies have shown the presence of large amounts of extracellular miRNAs outside EXOs and MVs, which are often associated with Argonaute (Ago) proteins. These miRNAs could result from the death of many cells, remaining stable in the extracellular environment thanks to binding with the Ago-2 protein [154]. miRNAs, specifically those incorporated in exosomes, are actively secreted and thus involved in unidirectional intracellular trafficking, causing phenotypic changes of the recipient cells [155]. EXO miRNAs can regulate gene expression at the translational and post-translational levels [156]. All body fluids are rich in exosomes and therefore in the miRNAs packed inside the vesicles. For this reason, exosomes are utilized as noninvasive biomarkers [157], especially in cancer prognosis. For example, EXO miR-451a, miR-21 and miR-4257 are altered in lung cancer and thus associated with tumor progression and poor prognosis [158].

The intercellular signaling and the structural stability and rigidity of exosomes is normally guaranteed by several components such as cholesterol, arachidonic acid, diglycerides, sphingolipids (i.e., sphingomyelin and ceramide), phospholipids, glycerophospholipids including PS, phosphatidylcholine (PC), phosphatidylinositol (PI), and phosphatidylethanolamine (PE), but also bioactive lipids such as prostaglandins and leukotrienes [159]. Exosomes have a lipid content comparable to that present in the parental cell [160], but sometimes even higher for specific lipids (PS, PC, PI, cholesterol). This latter aspect increases the rigidity of the exosome membrane.

There are not many studies on the molecular composition of MVs, but the presence of lipids, proteins and nucleic acids similar to those of exosomes has been identified. The proteomic cargo of MVs is characterized by various molecules, already mentioned in EXOs, including Alix, TSG101, the tetraspanins CD9, CD81, CD63, CD86, CD40, integrins, selectins, and glycoproteins, e.g., GPIb [85,161], but also matrix metalloproteinases (MMPs) [85,162] and cytoskeletal proteins, e.g., β-actin [163]. From a lipidomic point of view, MVs’ membranes are enriched with different lipids, mostly PC (59.2%), but also sphingomyelin (SM), PE, PS, cholesterol and diacylglycerol [164,165,166,167,168,169]. Instead, ApoBDs, based on their origin, are composed of a myriad of proteins such as heat shock proteins, e.g., HSPB6, RAB11A, Annexins, e.g., ANXA6 or ANXA5, histones and cytosolic proteins [168,170]. Moreover, these vesicles are rich in PS [171]. In addition to exosomes, both MVs and ApoBDs are also characterized by the presence of nucleic acids including DNA, mRNA, miRNA and rRNA [172,173].

### 2.3. EVs in Lung Homeostasis

Epithelia are characterized by a higher turnover rate in comparison to other tissue compartments, as old cells are continually replaced by new cells derived from differentiation into the staminal niche. However, as compared to other organs (e.g., skin, gut), in physiological conditions, lungs are characterized by a very slow cellular turnover [174]. It is well known that lungs are daily exposed to noxious stimuli such as environmental pollutants and microorganisms, which can give rise to an inflammatory process. When damaged, lungs are able to regenerate and repair its different tissues. In the “steady state”, the cell renewal is very low, while after injury a regenerative response takes place thanks to interactions between stem cells and the surrounding environment [175]. Lungs are characterized by regenerative processes that respond to a kind of “regionalization”. Indeed, in the proximal airways, the progenitor compartment is represented by basal cells that self-renew and, in the presence of tissue injury, can differentiate into different cytotypes such as secretory, goblet, ciliated and neuroendocrine cells so as to guarantee the integrity of airway structures. Apart from basal cells, secretory cells possess self-renewing properties and a good differentiative activity [176,177,178]. Alveoli, which are representative of the distal region, are composed of alveolar type 1 (AT1) and alveolar type 1 (AT2), with the latter having a staminal/progenitor behavior, which is particularly important both during the formation of alveoli and after cell damage in adult lungs [179,180,181]. In addition, during lung regeneration, the stromal components supporting the epithelial staminal niche, e.g., mesenchymal and immune cells, seems to be very important [182].

In recent years, it has been demonstrated that EVs regulate the lungs’ homeostasis as well as their functions [183,184,185,186]. Many types of respiratory cells can release EVs (MVs and EXOs), including alveolar epithelial cells, alveolar macrophages, pulmonary vascular endothelial cells, airway and vascular smooth muscle cells, fibroblasts, stromal cells, and immune cells [93]. Recently, it has been demonstrated that EVs derived from airway epithelial cells are fundamental for both the communication among different epithelial cells and the homeostasis of airways and alveoli. When airway epithelial cells take up EVs derived from different airway cell populations, many molecules with important roles in cell life, such as proteins and miRNAs, are differently expressed or regulated. Recently, Kadota et al. [187] published a paper concerning the role EVs in the modulation of cell signaling involved in IPF, a disease in which the lung parenchyma is deeply compromised, resulting in poor patient prognosis. When given intratracheally in a mouse model of bleomycin-induced lung fibrosis, bronchial epithelial cell-derived EVs can attenuate WNT signaling, thereby suppressing the differentiation of myofibroblasts and epithelial cell senescence. The antifibrotic activity of bronchial epithelial cell EVs is higher as compared to that of mesenchymal stem cell EVs. It is interesting to point out that this effect is partly due to the miRNA cargo, which is able to negatively regulate the WNT–TGF-β crosstalk. In addition to the attenuation of the profibrotic cell phenotype and experimental lung fibrosis in vivo, they observed a negative modulation of myofibroblast differentiation and cellular senescence in lung fibroblasts (LFs). These results indicate that the direct administration of bronchial epithelial cell-derived EVs into the trachea may be a potential therapeutic approach to treat IPF by targeting the TGF-β–WNT crosstalk. As described above, another important source of EVs are alveolar epithelial cells. In normal conditions, these structures are characterized by a very slow cellular turnover, but murine models have demonstrated how in the presence of cell damage, AT2 cells acquire significant regenerative properties characterized by their intense cell proliferation and differentiation into AT1 cells. It is well established that EVs represent the way by which AT2 cells communicate with one another in orchestrating the maintenance or repair of damaged alveoli. Quan et al. [188] showed that EXOs derived from A549 cells (an AT2 cell line) could induce the proliferation of AT2 cells via miR-371b-5p, but not differentiation into pluripotent-stem-cell (PSC)-derived cultures. Interestingly, in the same study, it was observed that both bleomycin-treated human-induced PSC (hiPSC)-differentiated AT2 cells (hiPSC-AT2) and primary human AT2 cells were able to secrete miR-371b-5p in EVs. These results are indicative of the potential role of miR-371b-5p as an inducer of re-epithelization in the alveolar niche when lung injury takes place. Another recent study concerning the possible beneficial role of AT2-cell-derived EXOs was carried out by Mitchell and colleagues using a murine model of hyperoxia-induced lung injury [189]. Exosomes were isolated from both hiPSCs and hiPSC-AT2 (alveolar-like phenotype) and bound to magnetic beads before being administrated to mice. It was demonstrated that in the mice that were administered the exosome/beads, the level of tissue injury was lower as compared with that observed in the control mice.

Currently, the roles of EVs derived from mesenchymal cells in epithelial stem/progenitor cells function during regeneration remain unclear. Some studies carried out using mesenchymal cells, such as LFs obtained from lung tissue of patients, have shown that EVs derived from these cells can induce abnormal epithelial regeneration. Haj-Salem et al. have demonstrated that LF-derived EXOs are central in the crosstalk between fibroblasts and epithelial cells [190]. Epithelial cells were challenged with EVs obtained from fibroblasts residing in the bronchi of severe eosinophilic asthma patients, and the effect was that they proliferated abnormally. This hyper-proliferation was due to the lower levels of the antiproliferative mediator TGF-β2.

In a study concerning IPF, Kadota et al. [191] have demonstrated that LF EXOs from patients can be taken up by epithelial cells, causing damage in their mitochondria as well as the induction of senescence. IPF LF EVs induce an overproduction of ROS by the mitochondria, which in turn causes mitochondrial damage, the activation of DNA damage response, and results in senescence. In IPF pathogenesis, it is very important to observe how the interstitial LFs differentiate into contractile myofibroblasts, which are characterized by a high proliferation and an excessive secretion of the extracellular matrix. It is also worth mentioning that some lipidic mediators, such as prostaglandins (PGs), can inhibit myofibroblast differentiation. Some researchers showed that the stimulation of primary human LFs with IL-1β inhibited the differentiation into contractile myofibroblasts of both themselves (autocrine signal) and adjacent naive LFs in co-cultures (paracrine signal). Interestingly, the same study demonstrated for the first time that the effects of the antifibrotic properties of activated fibroblasts on fibroblasts close to them are mediated by EXOs and other EVs, the content of which is represented by several PGs, including the antifibrotic PGE2.

Another important source of EVs in the lungs is represented by resident immune cells with multiple subclasses of dendritic cells, innate lymphoid cells and interstitial and alveolar macrophages. Macrophage-derived EVs can induce regenerative processes in lung epithelia. SOCS1 and SOCS3 (suppressors of cytokine signaling) are two anti-inflammatory mediators contained in EVs that are secreted by alveolar macrophages (AM). They inhibit Janus kinase–STAT signaling, which represents an important pathway activated by many cytokines [192]. Moreover, SOCS3 contained within human alveolar macrophage EVs can exert antitumoral effects and decrease the production of some cytokines involved in allergic response, such as IL-4, IL-13 and thymic stromal lymphopoietin, from airway epithelial cells [193]. Furthermore, in the presence of LPS, macrophages secrete EVs. Moreover, in the early phases of LPS stimulation, these EVs are mostly ABs, which contain miRNAs such as miR-221 and miR-222. Functionally, ABs produced upon the LPS stimulation of macrophages are able to induce the proliferation of malignant and/or normal lung epithelial cells. miR-221/222 deletion in ABs significantly reduces the AB-mediated proliferation, demonstrating that the AB-shuttling of miR-221/222 itself promotes cell growth [194]. Moreover, an important study was carried out with the purpose of evaluating the impact of some miRNAs on the fibrosis associated with IPF progression. Interestingly, the results of this study demonstrated that macrophages isolated from sputum are rich in exosomal miR-142-3p, and that macrophage-derived exosomes inhibit pulmonary fibrosis progression via the delivery of miR-142-3p [195]. It is noteworthy that macrophage EXOs exert an important role as promoters of epithelial cell maintenance.

Resident MSCs represent another important source of EVs in the lung. Several in vivo models have demonstrated the beneficial role of MSC-derived EVs and MVs in inflammatory conditions caused by bacteria and viruses [196,197,198,199]. Silva et al. [200] demonstrated that MSC-EVs were able to improve the alveolar–capillary barrier in experimental models of ARDS (acute respiratory distress syndrome). Even if it is still not clear how dysfunctional mitochondria contribute to the compromised alveolar–capillary barrier, this study demonstrates that MSC-EVs can restore the functionality of mitochondria. The dysfunctional mitochondria respiration, caused by stimulation with LPS of primary human distal lung epithelial cells, is restored by the transfer of mitochondria contained in MSC-EVs. Moreover, in a murine model of an LPS-injured lung, treatment with MSC-EVs reduced lung injury and restored mitochondrial respiration. In another study [201], it was demonstrated how the uptake of MSC-derived EXOs can down-regulate the expression of SAA3 (serum amyloid A3), an important mediator of the acute phase of inflammation. It is noteworthy to specify that this effect has been observed with EVs obtained from miR-30b-3p-overexpressing MSCs. A further demonstration of the anti-inflammatory activity of miRNAs contained within MSC-EVs has been obtained [202,203]. The treatment of murine bone-marrow-derived MSCs with hypoxia increased miR-21-5p concentration in MSC-EXOs, which in turn attenuated ischemia/reperfusion injury in a mouse lung [202].

It has been also demonstrated that autophagy may have an important role in tissue regeneration mediated by MSCs [203,204]. A study by Wei et al. [205] further elaborated on the relationship between autophagy, inflammation and lung damage as exerted by MSC-EXOs. MiR-377-3p contained in EXOs from human umbilical cord MSCs protected lungs from acute injury induced by LPS. This beneficial effect was due to the down-regulation of RPTOR (target regulatory-associated protein of mTOR) mediated by miR-377-3p.

### 2.4. Dysregulation of EVs’ Cargo in the Pathogenesis of COPD and CF

EVs, containing a specific cargo of lipids, proteins, metabolites and nucleic acids, regulate the intercellular communication in both health and disease, influencing physiological and pathological processes (i.e., immune responses [206], tissue repair [207,208], stem cell maintenance [209], cardiovascular diseases [210,211], neurodegeneration and demyelinating diseases [212,213], cancer [214] and inflammation [215]). EXOs can trigger signaling and thus release their content in target cells in different ways: (1) at the cellular level through the interaction of the EXO membrane ligands with specific membrane receptors of recipient cells; (2) by a mechanism in which the membrane proteins of the exosomes are cleaved by the proteases and act as ligands for the target cell receptors; (3) by the direct fusion of the EXO membrane with target cells, resulting in its content being secreted into the cytosol of the recipient cell [216]; (4) at the intracellular level via the process of EXO endocytosis (i.e., clathrin-mediated endocytosis, caveolin-mediated endocytosis, micropinocytosis and phagocytosis) [217] by different types of cells including gastric epithelial cells [218], macrophages [219], cardiomyocytes [220], etc.

EVs, released from almost all cell types in the lung, play an important role in the pathogenesis of chronic respiratory diseases such as COPD and CF [27,221]. They mediate intercellular communication in the lungs and hence are involved in pulmonary homeostasis [222] and cellular response to stimuli and lung disease [221,223]. A dysregulation of the protein, lipid and nucleic acid (especially miRNA) content of exosomes has been highlighted in respiratory diseases that also involve inflammation, especially following exposure to various stimuli [224,225,226]. Several studies have shown that bronchial epithelial cells, which normally regulate airway homeostasis, release more extracellular vesicles than other cells [224]. These EVs are characterized on the surface by some mucins (MUC1, MUC4 and MUC16) [223] that protect against pathogens [227], maintain the structure of the vesicles [223], and allow the interaction of the vesicles with the inhaled substances or with the receiving cells. Epithelial EVs transport pro-inflammatory cytokines and are rich in miRNA-210, whose levels further increase after exposure to cigarette smoke extract (CSE) [228]. Xu et al. showed that CSE increased the levels of miRNA-21 in the EVs released by human bronchial epithelial cells in COPD patients [229]. Moreover, the lengthy exposure to cigarette smoke extract increased cellular communication network factor 1 (flCCN1) levels in EVs, which regulated the lung homeostasis [230]. Alipoor et al. highlighted the importance of airway epithelial cell exposure to tobacco smoke extract in increasing the miRNA content of exosomes such as miRNA-101 and miRNA-144, which inhibit CFTR protein expression in the pathogenesis of COPD [225].

Lung resident macrophage-derived exosomes also play a pivotal role in inflammation, immune function and tissue injury in the lung tissue microenvironment [231]. Macrophage EXOs are enriched with miRNA-223, which is targeted to recipient cells by activating the differentiation of monocytes into macrophages [232], and in MHC II molecules, they are important for antigen presentation and immune activation [233,234]. It has been previously shown that CSE exposition increased the secretion of macrophage MVs with pro-inflammatory activity in lung diseases [235]. Li et al. showed that CSE caused an alteration of the content of macrophage-derived MVs, leading to an up-regulation of MMP14 [162]. Furthermore, Cordazzo et al. showed that mycobacterium infection also increased the protein content of alveolar-macrophage (AM)-derived exosomes, especially HSP-70 [235], with pro-inflammatory activity mediated by NK cell activation [236].

EVs released by endothelial cells, particularly circulating microparticles (EMPs), are increased in patients with several diseases such as COPD [237] or in response to various stresses such as cigarette smoke extract [238]. CSE reduced the trafficking of α1AT mediated by endothelial-cell-derived EVs to lung epithelial cells [239]. Numerous studies showed an increase in CD31+/CD42b- or CD31+CD62E+ EMPs [240,241,242,243] in COPD patients and in healthy smokers, which decreased in smokers who stopped smoking, suggesting the important role of EVs in inducing apoptosis and endothelial damage with poor repair capacity [240]. On the other hand, studies using mouse models investigated the EVs’ cargo following prolonged exposure over time to the cigarette smoke extract, discovering an increase in CD31+/42b- EMPs [244].

EVs also have great potential in CF lung disease. Exosomes derived from CF epithelial cells of the lung airways have a different protein content than the EVs released by healthy cells [245]. EVs mediate inflammation by regulating the migration and activation of neutrophilic leukocytes in the CF airways. This is allowed by the rich content of the CF airway bronchial-epithelial-cell (CFBE41o-)-derived exosomes in the integrin proteins, such as VCAM1 [245], with consequent migration of neutrophils to inflammatory sites [246], and in the ligand S100 A12 [245], which can bind the RAGE receptor placed on the surface of the receiving neutrophils, causing their activation [247].

**Figure 1:**
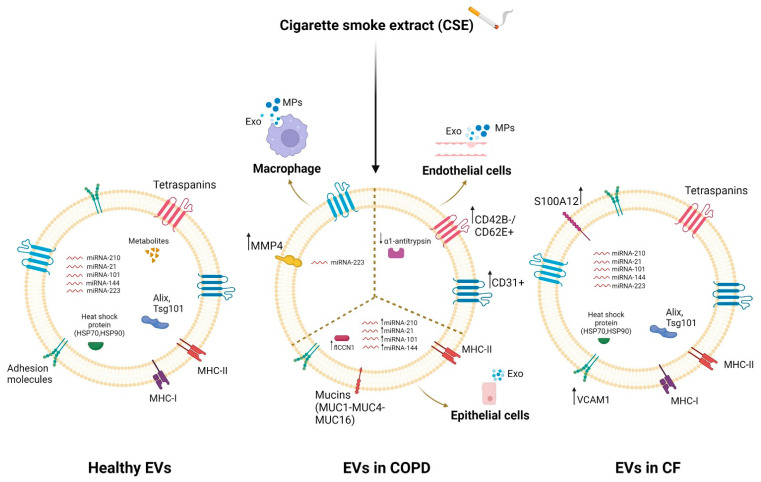
Molecular cargo of healthy EVs and vesicles released by various cell types in the pathogenesis of chronic obstructive pulmonary disease (EVs in COPD) and cystic fibrosis (EVs in CF). The increase in some vesicle’s components, involved in certain respiratory diseases, is indicated by an upward arrow. The exposure to various stimuli such as cigarette smoke extract leads to changes in the content of EVs released by the various cell types in the lung. In COPD, MMP4 and miRNA-223 levels increase in the vesicles released by macrophages; epithelial-cell-derived EVs show an overexpression of miRNA-101, miRNA-21, miRNA-210, miRNA-144 and an increase in the cellular communication network factor 1 (flCCN1) levels; endothelial cells secrete exosomes and microparticles (MPs) rich in CD31+/CD42b- or CD31+/CD62E+ and with reduced levels of α1-antitrypsin. In CF, the expression of the integrin protein VCAM1 and the ligand S100A12 is enhanced in the EVs released by the airway bronchial epithelial cells. Created with BioRender.com.

Differences in EVs’ cargoes between healthy individuals and CF/COPD patients are highlighted in Figure 1.

## 3. EVs and CF

Our research group assessed if the sputum from CF patients contained EVs [248]. These EVs were MPs (100–500 nm) and expressed CD66b (granulocytes, median value of 53.8%), CD11a (leukocytes, median value of 16.1%), and CD11b (monocyte/macrophages, median value of 0%). Thus, according to these data, the MPs were derived from granulocytes (activated or apoptotic), while the presence of monocyte macrophage MPs was not statistically significant. Considering the presence of MPs in the sputum of CF patients, we asked ourselves whether MPs could have a role in the pathophysiology of lung disease. We approached this question by injecting MPs into the lungs of Swiss mice, finding that they caused a massive infiltration of neutrophils in the lung parenchyma and perivascular/peribronchial regions. Interestingly, these neutrophil-derived MPs presented high LPS-like activity, likely as a consequence of the binding of shed LPS in CF airways [249].

A proteomic study on BALF of CF, primary ciliary dyskinesia (PCD) and asthmatic patients identified that PCD and CF respiratory exosomes displayed higher levels of both grancalcin and histones [250], which mediate important properties of neutrophils such as their ability to adhere to fibronectin, their degranulation, and the increase in their killing antimicrobial activity [251]. On the other hand, CF EXOs contained more inflammation-related proteins, such as neutrophil gelatinase-associated lipocalin (LCN2) and S100A12. LCN2 is stored in neutrophil secondary granules and can also be synthesized de novo by epithelial cells and macrophages in response to inflammatory stimuli, acting as a neutrophil chemoattractant [252]. S100A12 is a member of the S100 family of calgranulin small calcium-binding proteins, expressed predominantly in the cytosol of neutrophils and monocytes, and found elevated in CF BALF [253]. It supports neutrophil interactions with fibrinogen, their localization into inflammatory sites, and the expression of cytokines and chemokines via binding to its receptor RAGE (receptor for advanced glycation end products) [254,255]. EXOs collected from the CF patients’ airways were also characterized by high levels of superoxide dismutase (SOD2) and glutathione peroxidase 3 (GPX3), two proteins that can act synergistically in antioxidant defense, and peroxiredoxin 5 (PRDX5), all contributing to the regulation of airway inflammation.

The pathologic role of EVs in CF lung disease was confirmed by Useckaite et al. [245] in an in vitro study. A significantly higher concentration of EVs (50–250 nm, small EV subtype that potentially contains EXOs, small MPs and exomeres) were released from CF airway cell lines (CFBE41o- and CuFi-5) compared with the WT control cell lines (HBE41o- and NuLi-1). CFBE41o- EVs were taken up by healthy donor neutrophils, which increased their CD66 expression, increased myeloperoxidase activity and were stimulated to migrate via the interaction of RAGE on neutrophils and EV-derived S100A12.

More recently, Forrest and colleagues [256] showed that CF sputum EVs can activate naïve neutrophils, inducing both the exocytosis of their primary granule and their concomitant caspase-1 and IL-1β production. Moreover, EVs produced by activated neutrophils allow the delivery of active caspase-1 to primary tracheal epithelial cells which, in turn, activate the inflammasome and release IL-1α, IL-1β, and IL-18. Some studies have also shown the presence of pro-inflammatory EVs, with high protein content, in CF BALF [250] and sputum [249], which are involved in the activation and chemotaxis of neutrophils.

**Figure 2:**
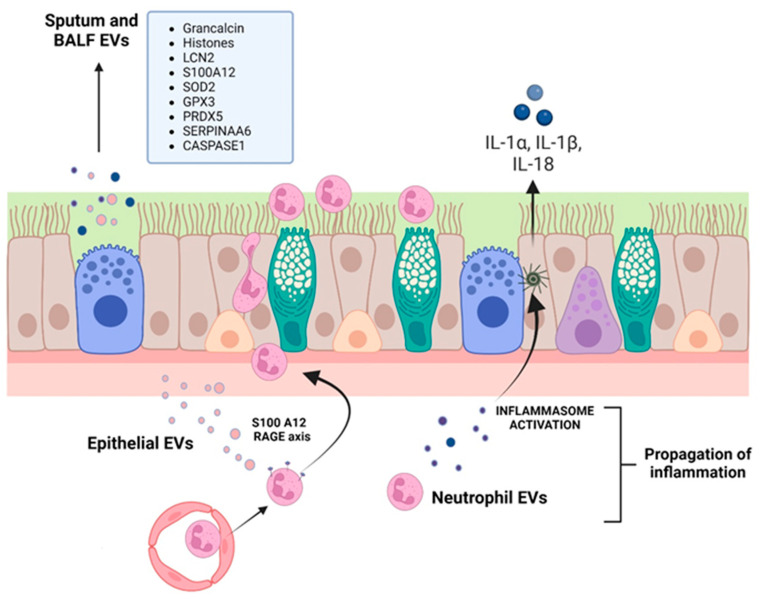
Involvement of EVs in CF inflammatory lung disease. EVs released by airway epithelial cells and enriched with S100A12 increase RAGE on blood neutrophils, which transmigrate and exudate into the CF airway lumen. On the other hand, neutrophil-derived EVs induce inflammasome activation in airway epithelial cells and the subsequent secretion of the cytokines Il-1α, IL-β and IL-18, thus propagating inflammation. All these EVs are then transported into the airway lumen and found in CF airway secretions (sputum, BALF). Created with BioRender.com.

Summarizing all these findings (Figure 2 and Table 1), it has been demonstrated that EVs produced by CFTR-mutated epithelial cells can activate neutrophils and vice versa, that is, neutrophil EVs can activate epithelial cells, namely their inflammasome (Figure 2). What has been a conundrum of CF lung disease, i.e., how a chronic disease is characterized by a neutrophil-dominated inflammation, a typical acute inflammatory cell type, can be partly explained by the secretion of two neutrophil-activating proteins (LCN2 and S100A12) at a distance from the initial lesion by EVs. Indeed, EVs produced in discrete areas of the bronchiolar tree may well induce a spreading of signaling (involving inflammasome) via uptake by resident, long-lived epithelial cells, but also in freshly recruited neutrophils.

## 4. EVs and COPD

Fujita and colleagues [228] investigated whether CSE modified epithelial EVs, thus considering their role as novel paracrine intercellular communication mediators during airway remodeling in COPD pathology. LFs took up EVs (50 to 150 nm, i.e., in the EXO range) secreted by immortalized BEAS-2B cells or primary HBECs, with EVs obtained from cigarette smoke extract (CSE)-treated HBECs promoting the expression of fibrotic markers in LFs, that is, collagen type I and α-smooth muscle actin (α-SMA). miR-210 was enriched in HBEC-derived EVs and promoted myofibroblast differentiation in LFs through the inhibition of autophagy protein ATG7. Thus, the inhibition of autophagy mediated by transferred EVs allowed the myofibroblast to differentiate in response to tobacco smoke. This process seems to be fundamental in airway remodeling in COPD pathogenesis.

In vitro and in vivo experiments have demonstrated that another miRNA, miR-21, is involved in bronchial epithelial cell-derived exosome (≤200 nm)-mediated airway remodeling [229]. The levels of miR-21 were higher when MRC-5 cells (lung fetal fibroblasts) were co-cultured with CSE-treated HBE cells, but not in the co-culture of MRC-5 cells and normal HBE cells. When MRC-5 cells were in the same co-culture conditions, an up-regulation of collagen I and α-SMA was observed. The transfection of HBE cells with an miR-21 inhibitor and the subsequent treatment with CSE did not exert any effect on α-SMA or collagen I expression. On the other hand, both the treatment of co-cultures with GW4869, an inhibitor of exosome generation, and the down-regulation of exosomal miR-21 in HBE cells had an inhibitory effect on the differentiation of myofibroblasts. To gain insight into the signaling pathway leading to fibroblast differentiation caused by exosomal miR-21, the role of HIF-1α, a transcription factor involved in fibrosis and myofibroblast differentiation [257], was investigated. The application of EXOs produced by CSE-treated HBE cells onto MRC-5 cells induced the up-regulation of HIF-1α. Consistently, the levels of pVHL, a factor related to HIF-1α stabilization, were decreased. The gene silencing of HIF-1α reduced the levels of α-SMA and collagen I that were induced by CSE-treated HBE cell EXOs. When CSE-treated HBE EXOs were applied to MRC-5 cells, it was possible to observe a change in the expression of pVHL, HIF-1α, α-SMA, and collagen I. Interestingly, the down-regulation of exosomal miR-21 blocked these changes in expression, but the gene silencing of pVHL decreased the effect of the miR-21 inhibitor. Finally, in a BALB/c mouse model of COPD obtained with an eight-week exposure to CS, when mice were injected with an antagomir-21 via the tail vein weekly, simultaneously with the first CS exposure in CS-exposed mice, the down-expression of miR-21 prevented changes in pulmonary function and attenuated inflammation and airway obstruction. Moreover, the expression of pVHL was improved when mice were treated with antagomir-21, whereas a contraction of HIF-1α levels was observed. Overall, these results highlight that CS can modify the cargo of exosomes and identify miR-21 produced in the bronchial epithelial cells as a key factor for the differentiation of myofibroblasts through the pVHL/HIF-1α signaling pathway. However, the results revealed that the levels of α-SMA and collagen I were only partly regulated by miR-21/pVH, probably as a consequence of the regulation of airway fibroblast differentiation by multiple pathways. Indeed, the TGF-β1/WNT and the PI3K/AKT pathways are involved in fibroblast differentiation and the up-regulation of collagen I/III in COPD, respectively [258,259]. It could be interesting to see whether exosomal miR-21 can also mediate myofibroblast differentiation through these other pathways. Furthermore, the role of exosomes in the dysfunctional crosstalk of the various cell types involved in airway remodeling (goblet cells, smooth muscles cells, and lung fibroblasts) needs to be clarified.

Emphysema is due to the destruction of alveolar tissues due to neutrophil influx into the airways and elastin degradation by a protease overwhelming of antiproteases, mainly α1-AT, and epithelial apoptosis. To study these pathological features, Moon and colleagues focused on the role of CCN1, also called Cyr61, a cysteine-rich, 38 kD secreted protein that is expressed by several cytotypes comprising airway epithelial cells and that induces IL-8 secretion by these cells [260]. There existed two forms of CCN1, including a full-length (fl)CCN1 that was cleaved into cCCN1 and found in the extracellular matrix [230]. However, the secretion pathway of the two forms differed: while the larger part of flCCN1 was identified in exosomes (as verified by size range (TEM) and Rab27a expression), cCCN1 was found in the supernatant of the conditioned medium as a soluble component. Secreted plasmin (upon CSE challenge) was responsible for the flCCN1 cleavage at the extracellular level. Only exosomal flCCN1was involved in the secretion of both IL-8 and VEGF by airway epithelial cells, while the soluble fraction (i.e., cCCN1) was mainly implicated in the secretion of MMP1. Integrins, particularly α_7_ chains, were implicated in the cCCN1-mediated MMP1 secretion. By using a mouse emphysema model, by which emphysematous changes occurred after six months of exposure to CS (but not at one month), it was observed that the BALF cCCN1 level was increased in samples collected from mice after six months of CS exposure but not from those after one month, showing that CCN1 levels correlated with the development of emphysema [230]. Overall, these data suggest that surface exosomal flCCN1 is cleaved by plasmin and cCCN1 interacts with integrin-α_7_ and activates the secretion of MMP1 in lung epithelial cells. A complex role for flCCN1 is also suggested by this study. flCCN1 may be well suitable to be a fundamental signaling molecule in lung homeostasis and pathophysiology, since it induces inflammatory responses via mediating IL-8 secretion and subsequent neutrophil enrollment, whereas, on the other hand, it may also have a role in sustaining the integrity of lung tissue by facilitating VEGF secretion. Interestingly, the progressive increase in cCNN1 during CS exposure induced MMP1 secretion while decreasing that of VEGF [230], implying a role for CCN1 in elevating MMP1 and down-regulating VEGF, alterations which have been well documented in inducing emphysema [261,262,263]. As concerning exosomes, another implication of this study is that the exosome-mediated secretion of flCCN1 potentially allows the propagation of “inflammatory” signals to distant portions of the lungs.

Besides epithelial cells, other cell types have been implicated in EV release and the pathophysiology of COPD, such as macrophages and endothelial cells. Macrophages are involved in emphysematous lung destruction [264,265] through their expression of a variety of proteases [264,266,267]. Li and colleagues [162] found that tobacco smoke extract induced macrophages to release MVs (isolated by ultracentrifugation at 100,000× *g*) which possess proteolytic activities owing to a single transmembrane MMP, namely MMP14, which is also known as the membrane type 1 MMP. Based on biochemical and morphological studies on THP-1 monocytes differentiated into macrophages and primary human monocyte-derived macrophages, it was demonstrated that the release of these MVs (approximately 1 μm by confocal microscopy) depends on dynamic, regulated steps that include the activation of the JNK and p38 MAPKs, the induction of cellular MMP14 via MAPK, the cleavage of pro-MMP14 into its active mature form, an increase in MMP14 into shedding plasma membrane blebs, and finally, caspase- and MAPK-dependent apoptosis and apoptotic blebbing. Further studies to explore the role of macrophage MVs in the damage of the lung extracellular matrix in COPD are warranted.

MPs were identified in the sputum of COPD patients and were released by different cell types, such endothelial cells, granulocytes, monocytes/macrophages, platelets/megakaryocytes, and red blood cells by flow cytometric analysis [268]. Notably, those derived from endothelial cells negatively correlated with forced expiratory volume in 1 s (FEV_1_), likely reflecting the increased apoptosis of endothelial cells [269] with the worsening of the clinical condition. The possible role of endothelial-cell-derived MPs was investigated by Lockett et al. [239], who searched for α1AT release in the lung across an intact pulmonary endothelial barrier. In primary rat pulmonary endothelial cell monolayers, it was shown that a nonclassical pathway of α1AT secretion was effectuated via the release of MPs (isolated at 100,000× *g*). Interestingly, CSE exposure reduced the levels of trans-cytosed α1AT, suggesting that CS may inhibit α1AT delivery into epithelial cells mediated by EVs secreted by endothelial cells, this process being fundamental in COPD pathogenesis.

More recently, a role for neutrophil EVs in emphysema was disclosed by Genschmer et al. [270] and Margaroli and colleagues [271]. In the first of these studies [270], it was found that EXOs (~100 nm by NTA) from activated neutrophils were enriched with surface neutrophil elastase (NE), which was enzymatically active and resistant to inhibition by α1AT. When exosomes were treated with purified human NE, their configuration changed, exposing the NE activity on their surface, implying that the loading of exosomes with NE occurs during the exocytosis of primary granules in the close proximity of released exosomes. Activated, but not quiescent, exosomes visibly destroyed the collagen fibrils via the integrin Mac-1 αM-I domain and bound to elastin fibers. In a model of murine COPD by intratracheal injection, exosomes from activated, but not quiescent, neutrophils caused alveolar enlargement, increased airway resistance, and right ventricular hypertrophy (RVH), compared to control mice. To determine the collagen disruption, the murine BALF was then measured for the quantity of acetyl-proline-glycine-proline (PGP), a product derived from collagen degradation, associated with ECM turnover and generally increased in lung secretions from COPD patient [272]. The PGP levels in the BALF of the activated-exosome-treated but not quiescent-exosome-treated animals were significantly increased. In mice treated with BALF CD66+ exosomes collected from COPD patients (~120 nm by NTA), it was possible to observe a marked alveolar enlargement and RVH. On the contrary, mice administered with pooled BALF exosomes from healthy controls did not present pathological changes. The majority of CD66b+ exosomes (96.1%) in COPD BALF expressed NE, whereas few (1.3%) of those collected from healthy, nonsmoking (NS) control BALF expressed NE, suggesting that COPD BALF exosomes principally induce alveolar enlargement by NE present on the CD66b+ population of exosomes (whereas NS CD66b+ purified BALF exosomes did not).

Margaroli et al. [271] extended these findings by implementing a mouse-to-mouse EV transfer model. Intratracheal administration of LPS in A/J mice allowed to determine the presence of proteolytically active α1AT-resistant neutrophil exosomal NE in BALF 24 h later. The administration of these EVs (EXO size range of 50–100 nm) in a single dose to A/J or C57BL/6 mice resulted in alveolar enlargement and a dose-response relationship, with significant effects observed with 1 × 10^6^ EVs and a robust response beginning at 1 × 10^7^ EVs. EV-mediated alveolar enlargement from a single 1 × 10^7^ EV dose lasted for at least three weeks, with the induction of low levels of inflammation at weeks one and two. The alveolar enlargement translated into local and systemic pathological changes, in that mice showed increased airway resistance, lower forced expiratory volume in 0.1 s (FEV0.1), and right ventricular hypertrophy one week after dosing. When EVs were isolated from NE-KO (Elane^–/–^) mice after the induction of airway inflammation via LPS, the alveolar enlargement effect was lost. The same result was obtained when the EV neutrophil-derived population was depleted via a bead-based pulldown method (Ly6G^+^).

**Figure 3:**
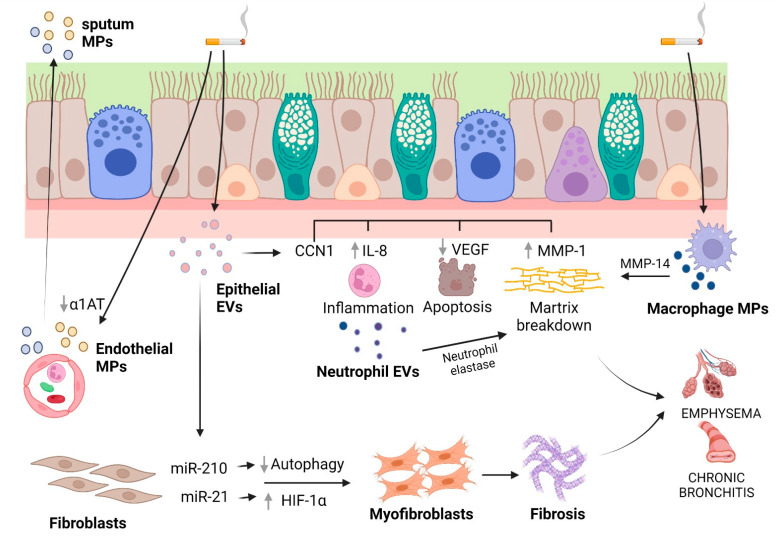
Involvement of EVs in COPD inflammatory lung disease and remodeling. Cigarette smoke induces EV release, which due to their miRNAs cargo cause reduced autophagy (miR210) or an increase in HIF-1a, both events leading to fibroblast differentiation in myofibroblasts and eventually lung fibrosis. Epithelial EVs induced by cigarette smoke are enriched with CCN1, which has various effects, such as IL-8 secretion (and neutrophil recruitment), decreased VEGF (and apoptosis), and increased MMP1 levels (and matrix breakdown). Matrix degradation is also due to macrophage MPs, whose release is triggered by smoke, and that are enriched with MMP-14. Endothelial-cell-derived MPs are enriched with α1AT, which could be down-regulated by smoke. They have been found also in the sputum of CF patients, together with those derived by circulating granulocytes, platelets, and red blood cells. Created with BioRender.com.

Summarizing all of these studies, a complex role of EVs in COPD pathogenesis emerges (Figure 3 and Table 1). Many cell types are involved, such as airway and respiratory epithelial cells, lung macrophages, endothelial cells and neutrophils. It is likely that EVs participate in many amplifying steps in COPD pathogenesis, including inflammation, tissue destruction and fibrosis.

**Table 1:** Pathogenic roles of EVs in CF and COPD.

Study	Ev Type	Lung Disease	Study Type	Pathogenic Outcomes
Porro et al., 2010 [248]	Microparticles (100–500 nm)	Cystic fibrosis	Patients	MPs were found in CF sputa and were mostly of granulocyte origin (CD66b^+^), while leukocytes (CD11a^+^) and monocyte/macrophages (CD11b^+^) MPs were less present.
Porro et al., 2013 [249]	Microparticles (100–500 nm)	Cystic fibrosis	In vivo, in mice	I.t. injection of sputum MPs obtained from a CF patient in acute conditions in Swiss mice induced peribronchial/perivascular infiltrates, similar to that obtained with LPS, whereas the inflammatory response was lower when MPs obtained from stable CF patients were injected.
Rollet-Cohen et al., 2018 [250]	Exosomes (20–150 nm)	Cystic fibrosis	In vivo, in patients	BALF exosomes CF patients were enriched with proteins implicated in neutrophil function, such as chemotaxis (LCN2, S100A12) and degranulation (grancalcin), antioxidant proteins (SOD2, GPX3, PRDX5), antiproteases (SERPINAA6), and those involved in the response to the chronic infectious challenge (histones, TOLLIP).
Useckaite et al., 2020 [245]	50–250 nm (exosomes, small microparticles and exomeres)	Cystic fibrosis	In vitro	Higher EV levels were found in CFBE41o- and CuFi-5 cells as well as in BALF of patients with CF. A significant increase in neutrophil chemotaxis was observed with CFBE41o-EVs as compared to control EVs.
Forrest et al., 2022 [256]	<400 nm	Cystic fibrosis	Ex vivo	EVs isolated from CF sputa were positive for active caspase 1, induced transmigration of neutrophils, which showed primary granule exocytosis and increase in intracellular active caspase-1. Neutrophil-derived EVs up-regulated caspase 1 in primary tracheal cells.
Li et al., 2013 [162]	Microparticles	COPD (emphysema)	In vitro	MVs released from TSE-exposed macrophages (THP-1 macrophages and hMDMs) carried substantial gelatinolytic and collagenolytic activities that could be predominantly attributed to a transmembrane MMP14. TSE induced MMP14 accumulation in small, circumscribed cell-surface domains and an increase in MMP14-enriched MPs. Activation of the JNK and p38 MAPKs and apoptosis were a requisite for MMP14-positive MVs release.
Moon et al., 2014 [230]	Exosomes (by TEM)	COPD (emphysema)	In vitro, in vivo	CSE increased the percentage of CCN1-positive exosome in bronchial Beas2B cells. IL-8 and VEGF secretion was increased by exosomal full-length (fl)CCN1. MMP-1 secretion was increased predominantly by secreted cleaved (c)CCN1. In C57BL/6 mice exposed to CS, emphysematous changes were observed after 6 months. The cCCN1 level in BALF was highly elevated in BALF obtained from mice after 6-month CS exposure but not from those after 1-month exposure.
Fujita et al., 2015 [228]	50–150 nm (exosomes)	COPD	In vitro	EVs derived from cigarette smoke extract-stimulated bronchial epithelial cells promoted lung fibroblasts to differentiate into myofibroblasts through miR-210 transfer and autophagy suppression.
Lacedonia et al., 2016 [268]	MPs	COPD	Patients	MPs were isolated from the sputum of mild to severe COPD patients and were positive for CD66b (granulocytes), CD235ab (erythrocytes), CD31 (platelets/endothelial cell adhesion molecules 1), CD41 (platelets/megakaryocytes), and CD11a (leukocytes). There was a negative correlation between CD31-MPs and FEV_1_, whereas CD66b-MPs were correlated with a worse COPD performance index.
Xu et al., 2018 [229]	≤200 nm (exosomes)	COPD	In vitro and in vivo	Higher levels of α-SMA and collagen 1 in MRC-5 cells (bronchial fibroblasts) exposed to exosomes derived from CSE-treated HBE cells. In MRC-5 cells, down-regulation of exosomal miR-21 blocked the exosome-induced myofibroblastdifferentiation phenotype as well as the increased and decrease levels of HIF-1α and pVHL respectively. In BLAB/c mice exposed to CS for 8 weeks, down-expression of miR-21 prevented changes in pulmonary function and attenuated inflammation and airway obstruction, restored pVHL expression and decreased HIF-1α levels.
Genschmer et al., 2019 [270]	~100 nm (exosomes)	COPD	In vitro, in vivo	Exosomes from activated neutrophils had considerably higher quantities of surface NE compared to quiescent exosomes. Exosomal NE was resistant to inhibition by α1AT. Activated, but not quiescent, exosomes destroyed the collagen fibrils over time. When exosomes were administered intratracheally into A/J mouse airways, activated but not quiescent neutrophil exosomes caused the hallmarks of COPD, alveolar enlargement, increased airway resistance, and RVH, compared to mice treated with PBS. Human COPD lung-derived CD63^+^/CD66b^+^ exosomes induced marked alveolar enlargement and RVH.
Margaroli et al., 2022 [271]	50–100 nm (exosomes)	COPD (emphysema)	In vivo	Higher levels of BALF neutrophil-derived exosomes in LPS-treated mice (A/J) than controls. Dose-dependent alveolar enlargement upon i.t. administration of LPS-derived exosomes in A/J and C57BL/6 mice.

α1AT: α1-antitrypsin; BALF: bronchoalveolar lavage fluid; CF: cystic fibrosis; COPD: chronic obstructive pulmonary disease; CS: cigarette smoke; CSE: cigarette smoke extract; EVs: extracellular vesicles; FEV_1_: forced expiratory volume in 1 s; hMDMs: human monocyte-derived macrophages; i.t.: intra-tracheal; MMP: metalloproteinase; MPs: microparticles; NE: neutrophil elastase; ROS: reactive oxygen species; RVH: right ventricular hypertrophy; TSE: tobacco smoke extract.

## 5. Role of EVs as Biomarkers in CF and COPD

The cargoes transported by EVs are cell-specific and can allow the use of EVs as diagnostic tools for a variety of pathologies, including cerebrovascular disease [273,274], diseases of the central nervous system [275], kidney [276], liver [277], lungs [16,278], and cancer [113,279,280,281,282]. The EV/EXO enrichment in several different biospecimens (serum/plasma, BALF, urine, cerebrospinal fluid, saliva, milk or other fluids), and the observations that their composition can discriminate a disease state vs. normal conditions, emphasize the role of EVs as novel circulating biomarkers [283]. Importantly, besides the fact that the physiological state and microenvironment of their cells of origin are reflected by EV subtypes, and that most cells secrete EVs with specific cargoes [284,285,286], their membrane protects internal contents, which are transferred from the parent cell cytosol to recipient cells after EV–cell fusion occurs. Although, in principle, the EVs’ phospholipid bilayer protects their content and makes them very stable in the extracellular environment, exogenously administered EVs show a relatively short half-life in circulation (in the range of tens of minutes), implying that EVs to be considered as reliable biomarkers should be dosed serially or differentially [287]. However, they possess long shelf stability [104].

Recent evidence, as explained above, points to the pathogenic role of EVs in CF and other neutrophilic airway diseases. EVs have been found in sputum and BALF samples obtained from people with CF, a finding compatible with the higher EV secretion from CF bronchial epithelial cells [245]. Although many inflammatory and remodeling biomarkers have been found and exploited in CF [288,289,290], only few of them have reached a clinical application, such as serum high sensitivity C-reactive protein and sputum neutrophil elastase [291]. Proteomic analyses have found many putative biomarkers in CF BALF, which may be involved in pathogenesis of lung disease. Rollet-Cohen and colleagues [250] detected 14 proteins that were differentially abundant amongst CF, asthma and PCD. A recent study highlighted that urine EXOs and their cargoes might be employed as noninvasive biomarkers, since these EXOs showed a clear separation in differentially expressed proteins between CF patients and healthy controls [292].

Another biomarker found in airway EXOs is prolyl endopeptidase (PE), an extracellular protease originating from collagen, which is a neutrophil chemoattractant [293] implied in the pathogenesis of neutrophilic chronic lung diseases [294,295]. Increased PE-EXO release was observed in airway epithelial cells via a TLR4-mediated mechanism, and the presence of protease-rich EXOs in the sputum specimens obtained from subjects with CF lung disease colonized by Gram-negative bacteria *P. aeruginosa* was determined [296]. Thus, PE-EXO may represent another biomarker of CF lung diseases.

However, this insightful exploration of EV cargoes in airway-derived fluid and urine has not translated to any biomarker exploitation for CF.

Altered protein and RNA profiles in COPD have been discovered by studying blood EVs [224]. Proteomic studies highlighted differentially expressed proteins in smokers, patients with COPD and nonsmokers. Sundar et al. [297] were the first to find that several plasma proteins (CD5 antigen-like, fibronectin, clusterin, gelsolin, hyaluronan-binding protein, apolipoprotein D, and EGF-containing fibulin-like ECM protein) were differentially enriched in smokers and patients with COPD compared to nonsmokers.

The study of circulating EMPs has been considered in the context of COPD disease pathomechanisms and biomarker discovery. Elevated levels of EMPs have been found in subjects with COPD [298,299,300,301]. Since not all those who smoke develop COPD, EMP quantitation and cargo analysis may have relevance for considering subjects at risk.

Sundar et al. [302] were also the first to explore plasma-derived EV small RNA cargoes from nonsmokers, smokers, and patients with COPD. EVs from nonsmokers (n = 6), smokers (n = 6) and patients with COPD (n = 8) were similar to each other both in the size distribution of EXOs and the total number of particles. These EVs were shown by RNA-seq analysis to be enriched with miRNAs, tRNAs, piRNAs, snRNAs, snoRNAs. Focusing on miRNAs as novel biomarkers in smokers and patients with COPD for further analysis, they found distinct miRNA profiles (up-regulated: miR-22-3p, miR-99a-5p, miR-151a-5p, miR-320b, miR-320d; and down-regulated: miR-335-5p, miR-628-3p, miR-887-5p and miR-937-3p) in COPD versus smokers or nonsmokers. Interestingly, a gene ontology analysis of nonsmokers vs. COPD comparison showed an enrichment of smoking-mediated pathways such as apoptosis, inflammation-related signaling, and oxidative stress response.

COPD patients experience a brisk reduction in airflow during acute exacerbation episodes, which are due to bacterial and/or viral infections, while specific causes could not be identified in about 30% of cases. Since EMPs may be involved in COPD pathogenesis, Takahashi and colleagues compared the levels of CD144+ MPs (VE-cadherin EMPs), CD31+/CD41- MPs (PECAM MPs), CD146 MPs (MCAM MPs) and CD62E+ MPs (E-selectin MPs) in stable COPD patients, patients with exacerbated COPD, and healthy non-COPD individuals [299]. It was found that VE-cadherin, PECAM and E-selectin EMP numbers were significantly higher in the patients with exacerbated COPD than in the stable COPD patients. Moreover, E-selectin EMP levels were significantly higher in COPD patients with frequent exacerbations than in those without (*p* < 0.001), and, interestingly, returned to levels similar to those of stable COPD patients without a history of frequent exacerbation on day 28, while the clinical exacerbation lasted until 14 days. A significant outcome of this study is that high E-selectin EMPs in patients with frequent exacerbations may predict COPD patients who may be susceptible to exacerbation.

In another study, circulating CD9+ EVs (i.e., exosomes) were found to be increased in patients with acute exacerbation vs. stable COPD patients vs. controls [303]. These EV levels correlated with systemic inflammatory markers (CRP, sTNFR1, IL-6 in plasma).

It can be concluded that plasma EVs may mediate important processes during inflammation in COPD-associated acute exacerbations that require further investigation.

## 6. Conclusions

The exploration of EVs of different sources in CF and COPD has determined that many of the mechanisms involved in pathobiology are mediated by MPs and EXOs. A piece of the puzzle which is missing is if the system is redundant or specific clues may be obtained from this knowledge. An insightful consideration about these issues may bring novel therapies which, in a favorable way, could lead to personalized medicines. For example, we do not know whether CF patients bearing a different class of mutations have EVs that behave in the same manner. Compounding with these studies, an effort towards the standardization of the isolation, characterization and functional assays for EVs isolated from airway cells should be brought forth. Finally, there is still room for understanding the richness of EVs in biological fluids (e.g., BALF, sputum) and their relevance as biomarkers. Prospective studies are necessary for the comprehension of EVs as theranostic (diagnostic and therapeutic) and prognostic biomarkers.

## References

[1] Grange C., Bussolati B. (2022). Extracellular vesicles in kidney disease. Nat. Rev. Nephrol..

[2] Ohayon L., Zhang X., Dutta P. (2021). The role of extracellular vesicles in regulating local and systemic inflammation in cardiovascular disease. Pharmacol. Res..

[3] Hwang H.S., Kim H., Han G., Lee J.W., Kim K., Kwon I.C., Yang Y., Kim S.H. (2021). Extracellular Vesicles as Potential Therapeutics for Inflammatory Diseases. Int. J. Mol. Sci..

[4] Mabrouk M., Guessous F., Naya A., Merhi Y., Zaid Y. (2022). The Pathophysiological Role of Platelet-Derived Extracellular Vesicles. Semin. Thromb. Hemost..

[5] Liu G., Yin X.M. (2022). The Role of Extracellular Vesicles in Liver Pathogenesis. Am. J. Pathol..

[6] Vandendriessche C., Bruggeman A., Van Cauwenberghe C., Vandenbroucke R.E. (2020). Extracellular Vesicles in Alzheimer’s and Parkinson’s Disease: Small Entities with Large Consequences. Cells.

[7] Trotta T., Panaro M.A., Cianciulli A., Mori G., Di Benedetto A., Porro C. (2018). Microglia-derived extracellular vesicles in Alzheimer’s Disease: A double-edged sword. Biochem. Pharmacol..

[8] Shah R., Patel T., Freedman J.E. (2018). Circulating Extracellular Vesicles in Human Disease. N. Engl. J. Med..

[9] Kubo H. (2018). Extracellular Vesicles in Lung Disease. Chest.

[10] Kato T., Vykoukal J.V., Fahrmann J.F., Hanash S. (2021). Extracellular Vesicles in Lung Cancer: Prospects for Diagnostic and Therapeutic Applications. Cancers (Basel).

[11] Liu Y., Luo F., Wang B., Li H., Xu Y., Liu X., Shi L., Lu X., Xu W., Lu L. (2016). STAT3-regulated exosomal miR-21 promotes angiogenesis and is involved in neoplastic processes of transformed human bronchial epithelial cells. Cancer Lett..

[12] Li X., Wang S., Zhu R., Li H., Han Q., Zhao R.C. (2016). Lung tumor exosomes induce a pro-inflammatory phenotype in mesenchymal stem cells via NFkappaB-TLR signaling pathway. J. Hematol. Oncol..

[13] Huang S.H., Li Y., Zhang J., Rong J., Ye S. (2013). Epidermal growth factor receptor-containing exosomes induce tumor-specific regulatory T cells. Cancer Invest..

[14] Rahman M.A., Barger J.F., Lovat F., Gao M., Otterson G.A., Nana-Sinkam P. (2016). Lung cancer exosomes as drivers of epithelial mesenchymal transition. Oncotarget.

[15] Kaplan R.N., Riba R.D., Zacharoulis S., Bramley A.H., Vincent L., Costa C., MacDonald D.D., Jin D.K., Shido K., Kerns S.A. (2005). VEGFR1-positive haematopoietic bone marrow progenitors initiate the pre-metastatic niche. Nature.

[16] Makiguchi T., Yamada M., Yoshioka Y., Sugiura H., Koarai A., Chiba S., Fujino N., Tojo Y., Ota C., Kubo H. (2016). Serum extracellular vesicular miR-21-5p is a predictor of the prognosis in idiopathic pulmonary fibrosis. Respir. Res..

[17] Tahyra A.S.C., Calado R.T., Almeida F. (2022). The Role of Extracellular Vesicles in COVID-19 Pathology. Cells.

[18] Xia X., Yuan P., Liu Y., Wang Y., Cao W., Zheng J.C. (2021). Emerging roles of extracellular vesicles in COVID-19, a double-edged sword?. Immunology.

[19] Moon H.G., Cao Y., Yang J., Lee J.H., Choi H.S., Jin Y. (2015). Lung epithelial cell-derived extracellular vesicles activate macrophage-mediated inflammatory responses via ROCK1 pathway. Cell Death Dis..

[20] Lee H., Zhang D., Laskin D.L., Jin Y. (2018). Functional Evidence of Pulmonary Extracellular Vesicles in Infectious and Noninfectious Lung Inflammation. J. Immunol..

[21] Wang J., Chen S., Bihl J. (2020). Exosome-Mediated Transfer of ACE2 (Angiotensin-Converting Enzyme 2) from Endothelial Progenitor Cells Promotes Survival and Function of Endothelial Cell. Oxid. Med. Cell Longev..

[22] Gunasekaran M., Bansal S., Ravichandran R., Sharma M., Perincheri S., Rodriguez F., Hachem R., Fisher C.E., Limaye A.P., Omar A. (2020). Respiratory viral infection in lung transplantation induces exosomes that trigger chronic rejection. J. Heart Lung Transplant..

[23] Dwivedi V., Yaniv K., Sharon M. (2021). Beyond cells: The extracellular circulating 20S proteasomes. Biochim. Biophys. Acta Mol. Basis Dis..

[24] Hassanpour M., Rezaie J., Nouri M., Panahi Y. (2020). The role of extracellular vesicles in COVID-19 virus infection. Infect. Genet. Evol..

[25] Balbi C., Burrello J., Bolis S., Lazzarini E., Biemmi V., Pianezzi E., Burrello A., Caporali E., Grazioli L.G., Martinetti G. (2021). Circulating extracellular vesicles are endowed with enhanced procoagulant activity in SARS-CoV-2 infection. EBioMedicine.

[26] Puhm F., Flamand L., Boilard E. (2022). Platelet extracellular vesicles in COVID-19: Potential markers and makers. J. Leukoc. Biol..

[27] Trappe A., Donnelly S.C., McNally P., Coppinger J.A. (2021). Role of extracellular vesicles in chronic lung disease. Thorax.

[28] CFTR2. Clinical and Functional Translation of CFTR. https://cftr2.org/.

[29] Lopes-Pacheco M. (2020). CFTR Modulators: The Changing Face of Cystic Fibrosis in the Era of Precision Medicine. Front. Pharmacol..

[30] Cheng S.H., Gregory R.J., Marshall J., Paul S., Souza D.W., White G.A., O’Riordan C.R., Smith A.E. (1990). Defective intracellular transport and processing of CFTR is the molecular basis of most cystic fibrosis. Cell.

[31] Jensen T.J., Loo M.A., Pind S., Williams D.B., Goldberg A.L., Riordan J.R. (1995). Multiple proteolytic systems, including the proteasome, contribute to CFTR process. Cell.

[32] Dalemans W., Barbry P., Champigny G., Jallat S., Dott K., Dreyer D., Crystal R.G., Pavirani A., Lecocq J.-P., Lazdunski M. (1991). Alterated chloride ion channel kinetics associated with the DF 508 cystic fibrosis mutation. Nature.

[33] Haws C.M., Nepomuceno I.B., Krouse M.E., Wakelee H., Law T., Xia Y., Nguyen H., Wine J.J. (1996). Delta F508-CFTR channels: Kinetics, activation by forskolin, and potentiation by xanthines. Am. J. Physiol..

[34] Sharma M., Pampinella F., Nemes C., Benharouga M., So J., Du K., Bache K.G., Papsin B., Zerangue N., Stenmark H. (2004). Misfolding diverts CFTR from recycling to degradation: Quality control at early endosomes. J. Cell Biol..

[35] Swiatecka-Urban A., Brown A., Moreau-Marquis S., Renuka J., Coutermarsh B., Barnaby R., Karlson K.H., Flotte T.R., Fukuda M., Langford G.M. (2005). The short apical membrane half-life of rescued {Delta}F508-cystic fibrosis transmembrane conductance regulator (CFTR) results from accelerated endocytosis of {Delta}F508-CFTR in polarized human airway epithelial cells. J. Biol. Chem..

[36] Okiyoneda T., Barriere H., Bagdany M., Rabeh W.M., Du K., Hohfeld J., Young J.C., Lukacs G.L. (2010). Peripheral protein quality control removes unfolded CFTR from the plasma membrane. Science.

[37] Abrami M., Ascenzioni F., Di Domenico E.G., Maschio M., Ventura A., Confalonieri M., Di Gioia S., Conese M., Dapas B., Grassi G. (2018). A novel approach based on low-field NMR for the detection of the pathological components of sputum in cystic fibrosis patients. Magn. Reson. Med..

[38] Gibson R.L., Burns J.L., Ramsey B.W. (2003). Pathophysiology and management of pulmonary infections in cystic fibrosis. Am. J. Respir. Crit. Care Med..

[39] Shteinberg M., Haq I.J., Polineni D., Davies J.C. (2021). Cystic fibrosis. Lancet.

[40] Conese M., Castellani S., D’Oria S., di Gioia S., Montemurro P., Khajah M.A. (2017). Role of Neutrophils in Cystic Fibrosis Lung Disease. Role of Neutrophils in Disease Pathogenesis.

[41] Laval J., Ralhan A., Hartl D. (2016). Neutrophils in cystic fibrosis. Biol. Chem..

[42] Conese M., Copreni E., Di Gioia S., De Rinaldis P., Fumarulo R. (2003). Neutrophil recruitment and airway epithelial cell involvement in chronic cystic fibrosis lung disease. J. Cyst. Fibros..

[43] Hartl D., Gaggar A., Bruscia E., Hector A., Marcos V., Jung A., Greene C., McElvaney G., Mall M., Doring G. (2012). Innate immunity in cystic fibrosis lung disease. J. Cyst. Fibros..

[44] Bruscia E.M., Bonfield T.L. (2016). Cystic Fibrosis Lung Immunity: The Role of the Macrophage. J. Innate Immun..

[45] Laselva O., Guerra L., Castellani S., Favia M., Di Gioia S., Conese M. (2022). Small-molecule drugs for cystic fibrosis: Where are we now?. Pulm. Pharmacol. Ther..

[46] Rabe K.F., Watz H. (2017). Chronic obstructive pulmonary disease. Lancet.

[47] Hogg J.C., Chu F., Utokaparch S., Woods R., Elliott W.M., Buzatu L., Cherniack R.M., Rogers R.M., Sciurba F.C., Coxson H.O. (2004). The nature of small-airway obstruction in chronic obstructive pulmonary disease. N. Engl. J. Med..

[48] American Thoracic S., European Respiratory S. (2003). American Thoracic Society/European Respiratory Society statement: Standards for the diagnosis and management of individuals with alpha-1 antitrypsin deficiency. Am. J. Respir. Crit. Care Med..

[49] Fucarino A., Pitruzzella A., Burgio S., Zarcone M.C., Modica D.M., Cappello F., Bucchieri F. (2021). Extracellular Vesicles in Airway Homeostasis and Pathophysiology. Appl. Sci..

[50] Fischer B.M., Pavlisko E., Voynow J.A. (2011). Pathogenic triad in COPD: Oxidative stress, protease-antiprotease imbalance, and inflammation. Int. J. Chron. Obstruct. Pulmon. Dis..

[51] Rab A., Rowe S.M., Raju S.V., Bebok Z., Matalon S., Collawn J.F. (2013). Cigarette smoke and CFTR: Implications in the pathogenesis of COPD. Am. J. Physiol. Lung Cell Mol. Physiol..

[52] Cantin A.M. (2016). Cystic Fibrosis Transmembrane Conductance Regulator. Implications in Cystic Fibrosis and Chronic Obstructive Pulmonary Disease. Ann. Am. Thorac. Soc..

[53] Friedlander A.L., Lynch D., Dyar L.A., Bowler R.P. (2007). Phenotypes of chronic obstructive pulmonary disease. COPD.

[54] Woodruff P.G., Agusti A., Roche N., Singh D., Martinez F.J. (2015). Current concepts in targeting chronic obstructive pulmonary disease pharmacotherapy: Making progress towards personalised management. Lancet.

[55] Raju S.V., Solomon G.M., Dransfield M.T., Rowe S.M. (2016). Acquired Cystic Fibrosis Transmembrane Conductance Regulator Dysfunction in Chronic Bronchitis and Other Diseases of Mucus Clearance. Clin. Chest Med..

[56] Janoff A. (1985). Elastases and emphysema. Current assessment of the protease-antiprotease hypothesis. Am. Rev. Respir. Dis..

[57] Janoff A., Carp H., Lee D.K., Drew R.T. (1979). Cigarette smoke inhalation decreases alpha 1-antitrypsin activity in rat lung. Science.

[58] Petrache I., Fijalkowska I., Medler T.R., Skirball J., Cruz P., Zhen L., Petrache H.I., Flotte T.R., Tuder R.M. (2006). alpha-1 antitrypsin inhibits caspase-3 activity, preventing lung endothelial cell apoptosis. Am. J. Pathol..

[59] Petrache I., Fijalkowska I., Zhen L., Medler T.R., Brown E., Cruz P., Choe K.H., Taraseviciene-Stewart L., Scerbavicius R., Shapiro L. (2006). A novel antiapoptotic role for alpha1-antitrypsin in the prevention of pulmonary emphysema. Am. J. Respir. Crit. Care Med..

[60] Chung K.F. (2001). Cytokines in chronic obstructive pulmonary disease. Eur. Respir. J. Suppl..

[61] Churg A., Wang R.D., Tai H., Wang X., Xie C., Wright J.L. (2004). Tumor necrosis factor-alpha drives 70% of cigarette smoke-induced emphysema in the mouse. Am. J. Respir. Crit. Care Med..

[62] Churg A., Zhou S., Wang X., Wang R., Wright J.L. (2009). The role of interleukin-1beta in murine cigarette smoke-induced emphysema and small airway remodeling. Am. J. Respir. Cell Mol. Biol..

[63] Shi J., Li H., Yuan C., Luo M., Wei J., Liu X. (2018). Cigarette Smoke-Induced Acquired Dysfunction of Cystic Fibrosis Transmembrane Conductance Regulator in the Pathogenesis of Chronic Obstructive Pulmonary Disease. Oxid. Med. Cell Longev..

[64] Clunes L.A., Davies C.M., Coakley R.D., Aleksandrov A.A., Henderson A.G., Zeman K.L., Worthington E.N., Gentzsch M., Kreda S.M., Cholon D. (2012). Cigarette smoke exposure induces CFTR internalization and insolubility, leading to airway surface liquid dehydration. FASEB J..

[65] Rasmussen J.E., Sheridan J.T., Polk W., Davies C.M., Tarran R. (2014). Cigarette smoke-induced Ca2+ release leads to cystic fibrosis transmembrane conductance regulator (CFTR) dysfunction. J. Biol. Chem..

[66] Cantin A.M., Hanrahan J.W., Bilodeau G., Ellis L., Dupuis A., Liao J., Zielenski J., Durie P. (2006). Cystic fibrosis transmembrane conductance regulator function is suppressed in cigarette smokers. Am. J. Respir. Crit. Care Med..

[67] Marklew A.J., Patel W., Moore P.J., Tan C.D., Smith A.J., Sassano M.F., Gray M.A., Tarran R. (2019). Cigarette Smoke Exposure Induces Retrograde Trafficking of CFTR to the Endoplasmic Reticulum. Sci. Rep..

[68] Raju S.V., Jackson P.L., Courville C.A., McNicholas C.M., Sloane P.A., Sabbatini G., Tidwell S., Tang L.P., Liu B., Fortenberry J.A. (2013). Cigarette smoke induces systemic defects in cystic fibrosis transmembrane conductance regulator function. Am. J. Respir. Crit. Care Med..

[69] Hassan F., Xu X., Nuovo G., Killilea D.W., Tyrrell J., Da Tan C., Tarran R., Diaz P., Jee J., Knoell D. (2014). Accumulation of metals in GOLD4 COPD lungs is associated with decreased CFTR levels. Respir. Res..

[70] Raju S.V., Lin V.Y., Liu L., McNicholas C.M., Karki S., Sloane P.A., Tang L., Jackson P.L., Wang W., Wilson L. (2017). The Cystic Fibrosis Transmembrane Conductance Regulator Potentiator Ivacaftor Augments Mucociliary Clearance Abrogating Cystic Fibrosis Transmembrane Conductance Regulator Inhibition by Cigarette Smoke. Am. J. Respir. Cell Mol. Biol..

[71] Ni I., Ji C., Vij N. (2015). Second-hand cigarette smoke impairs bacterial phagocytosis in macrophages by modulating CFTR dependent lipid-rafts. PLoS ONE.

[72] Rowe S.M., Jones I., Dransfield M.T., Haque N., Gleason S., Hayes K.A., Kulmatycki K., Yates D.P., Danahay H., Gosling M. (2020). Efficacy and Safety of the CFTR Potentiator Icenticaftor (QBW251) in COPD: Results from a Phase 2 Randomized Trial. Int. J. Chron. Obstruct. Pulmon. Dis..

[73] Solomon G.M., Hathorne H., Liu B., Raju S.V., Reeves G., Acosta E.P., Dransfield M.T., Rowe S.M. (2016). Pilot evaluation of ivacaftor for chronic bronchitis. Lancet Respir. Med..

[74] Solomon G.M., Fu L., Rowe S.M., Collawn J.F. (2017). The therapeutic potential of CFTR modulators for COPD and other airway diseases. Curr. Opin. Pharmacol..

[75] Harding C.V., Heuser J.E., Stahl P.D. (2013). Exosomes: Looking back three decades and into the future. J. Cell Biol..

[76] Deatherage B.L., Cookson B.T. (2012). Membrane vesicle release in bacteria, eukaryotes, and archaea: A conserved yet underappreciated aspect of microbial life. Infect. Immun..

[77] Doyle L.M., Wang M.Z. (2019). Overview of Extracellular Vesicles, Their Origin, Composition, Purpose, and Methods for Exosome Isolation and Analysis. Cells.

[78] EL Andaloussi S., Mager I., Breakefield X.O., Wood M.J. (2013). Extracellular vesicles: Biology and emerging therapeutic opportunities. Nat. Rev. Drug Discov..

[79] Akers J.C., Gonda D., Kim R., Carter B.S., Chen C.C. (2013). Biogenesis of extracellular vesicles (EV): Exosomes, microvesicles, retrovirus-like vesicles, and apoptotic bodies. J. Neurooncol..

[80] Thery C., Witwer K.W., Aikawa E., Alcaraz M.J., Anderson J.D., Andriantsitohaina R., Antoniou A., Arab T., Archer F., Atkin-Smith G.K. (2018). Minimal information for studies of extracellular vesicles 2018 (MISEV2018): A position statement of the International Society for Extracellular Vesicles and update of the MISEV2014 guidelines. J. Extracell. Vesicles.

[81] Thery C., Boussac M., Veron P., Ricciardi-Castagnoli P., Raposo G., Garin J., Amigorena S. (2001). Proteomic analysis of dendritic cell-derived exosomes: A secreted subcellular compartment distinct from apoptotic vesicles. J. Immunol..

[82] Cocucci E., Racchetti G., Meldolesi J. (2009). Shedding microvesicles: Artefacts no more. Trends Cell Biol..

[83] Tkach M., Thery C. (2016). Communication by Extracellular Vesicles: Where We Are and Where We Need to Go. Cell.

[84] Muralidharan-Chari V., Clancy J.W., Sedgwick A., D’Souza-Schorey C. (2010). Microvesicles: Mediators of extracellular communication during cancer progression. J. Cell Sci..

[85] Heijnen H.F., Schiel A.E., Fijnheer R., Geuze H.J., Sixma J.J. (1999). Activated platelets release two types of membrane vesicles: Microvesicles by surface shedding and exosomes derived from exocytosis of multivesicular bodies and alpha-granules. Blood.

[86] Stoorvogel W., Kleijmeer M.J., Geuze H.J., Raposo G. (2002). The biogenesis and functions of exosomes. Traffic.

[87] Keller S., Sanderson M.P., Stoeck A., Altevogt P. (2006). Exosomes: From biogenesis and secretion to biological function. Immunol. Lett..

[88] Crescitelli R., Lasser C., Jang S.C., Cvjetkovic A., Malmhall C., Karimi N., Hoog J.L., Johansson I., Fuchs J., Thorsell A. (2020). Subpopulations of extracellular vesicles from human metastatic melanoma tissue identified by quantitative proteomics after optimized isolation. J. Extracell. Vesicles.

[89] Zhang Y., Liu Y., Liu H., Tang W.H. (2019). Exosomes: Biogenesis, biologic function and clinical potential. Cell Biosci..

[90] Hessvik N.P., Llorente A. (2018). Current knowledge on exosome biogenesis and release. Cell. Mol. Life Sci..

[91] Joshi B.S., de Beer M.A., Giepmans B.N.G., Zuhorn I.S. (2020). Endocytosis of Extracellular Vesicles and Release of Their Cargo from Endosomes. ACS Nano.

[92] Zhang Y., Bi J., Huang J., Tang Y., Du S., Li P. (2020). Exosome: A Review of Its Classification, Isolation Techniques, Storage, Diagnostic and Targeted Therapy Applications. Int. J. Nanomed..

[93] Fujita Y., Kosaka N., Araya J., Kuwano K., Ochiya T. (2015). Extracellular vesicles in lung microenvironment and pathogenesis. Trends. Mol. Med..

[94] Frydrychowicz M., Kolecka-Bednarczyk A., Madejczyk M., Yasar S., Dworacki G. (2015). Exosomes—Structure, biogenesis and biological role in non-small-cell lung cancer. Scand. J. Immunol..

[95] Colombo M., Moita C., van Niel G., Kowal J., Vigneron J., Benaroch P., Manel N., Moita L.F., Thery C., Raposo G. (2013). Analysis of ESCRT functions in exosome biogenesis, composition and secretion highlights the heterogeneity of extracellular vesicles. J. Cell Sci..

[96] Mathivanan S., Simpson R.J. (2009). ExoCarta: A compendium of exosomal proteins and RNA. Proteomics.

[97] Schoneberg J., Lee I.H., Iwasa J.H., Hurley J.H. (2017). Reverse-topology membrane scission by the ESCRT proteins. Nat. Rev. Mol. Cell Biol..

[98] Hurley J.H., Hanson P.I. (2010). Membrane budding and scission by the ESCRT machinery: It’s all in the neck. Nat. Rev. Mol. Cell Biol..

[99] Willms E., Cabanas C., Mager I., Wood M.J.A., Vader P. (2018). Extracellular Vesicle Heterogeneity: Subpopulations, Isolation Techniques, and Diverse Functions in Cancer Progression. Front. Immunol..

[100] Yokoi A., Ochiya T. (2021). Exosomes and extracellular vesicles: Rethinking the essential values in cancer biology. Semin. Cancer Biol..

[101] Kowal J., Arras G., Colombo M., Jouve M., Morath J.P., Primdal-Bengtson B., Dingli F., Loew D., Tkach M., Thery C. (2016). Proteomic comparison defines novel markers to characterize heterogeneous populations of extracellular vesicle subtypes. Proc. Natl. Acad. Sci. USA.

[102] Minciacchi V.R., You S., Spinelli C., Morley S., Zandian M., Aspuria P.J., Cavallini L., Ciardiello C., Reis Sobreiro M., Morello M. (2015). Large oncosomes contain distinct protein cargo and represent a separate functional class of tumor-derived extracellular vesicles. Oncotarget.

[103] Kalluri R., LeBleu V.S. (2020). The biology, function, and biomedical applications of exosomes. Science.

[104] Zhou B., Xu K., Zheng X., Chen T., Wang J., Song Y., Shao Y., Zheng S. (2020). Application of exosomes as liquid biopsy in clinical diagnosis. Signal Transduct. Target. Ther..

[105] Zhang H., Freitas D., Kim H.S., Fabijanic K., Li Z., Chen H., Mark M.T., Molina H., Martin A.B., Bojmar L. (2018). Identification of distinct nanoparticles and subsets of extracellular vesicles by asymmetric flow field-flow fractionation. Nat. Cell Biol..

[106] Witwer K.W., Buzas E.I., Bemis L.T., Bora A., Lasser C., Lotvall J., Nolte-’t Hoen E.N., Piper M.G., Sivaraman S., Skog J. (2013). Standardization of sample collection, isolation and analysis methods in extracellular vesicle research. J. Extracell. Vesicles.

[107] Zhao Z., Wijerathne H., Godwin A.K., Soper S.A. (2021). Isolation and analysis methods of extracellular vesicles (EVs). Extracell. Vesicles Circ. Nucl. Acids.

[108] Gardiner C., Di Vizio D., Sahoo S., Thery C., Witwer K.W., Wauben M., Hill A.F. (2016). Techniques used for the isolation and characterization of extracellular vesicles: Results of a worldwide survey. J. Extracell. Vesicles.

[109] Wu Y., Deng W., Klinke D.J. (2015). Exosomes: Improved methods to characterize their morphology, RNA content, and surface protein biomarkers. Analyst.

[110] Guan S., Li Q., Liu P., Xuan X., Du Y. (2014). Umbilical cord blood-derived dendritic cells loaded with BGC823 tumor antigens and DC-derived exosomes stimulate efficient cytotoxic T-lymphocyte responses and antitumor immunity in vitro and in vivo. Cent. Eur. J. Immunol..

[111] Lazaro-Ibanez E., Lasser C., Shelke G.V., Crescitelli R., Jang S.C., Cvjetkovic A., Garcia-Rodriguez A., Lotvall J. (2019). DNA analysis of low- and high-density fractions defines heterogeneous subpopulations of small extracellular vesicles based on their DNA cargo and topology. J. Extracell. Vesicles.

[112] Lasser C., Jang S.C., Lotvall J. (2018). Subpopulations of extracellular vesicles and their therapeutic potential. Mol. Aspects Med..

[113] Kalluri R. (2016). The biology and function of exosomes in cancer. J. Clin. Investig..

[114] Mathieu M., Martin-Jaular L., Lavieu G., Thery C. (2019). Specificities of secretion and uptake of exosomes and other extracellular vesicles for cell-to-cell communication. Nat. Cell Biol..

[115] Bebelman M.P., Smit M.J., Pegtel D.M., Baglio S.R. (2018). Biogenesis and function of extracellular vesicles in cancer. Pharmacol. Ther..

[116] Palviainen M., Saari H., Karkkainen O., Pekkinen J., Auriola S., Yliperttula M., Puhka M., Hanhineva K., Siljander P.R. (2019). Metabolic signature of extracellular vesicles depends on the cell culture conditions. J. Extracell. Vesicles.

[117] Shaba E., Vantaggiato L., Governini L., Haxhiu A., Sebastiani G., Fignani D., Grieco G.E., Bergantini L., Bini L., Landi C. (2022). Multi-Omics Integrative Approach of Extracellular Vesicles: A Future Challenging Milestone. Proteomes.

[118] Nirujogi T.S., Kotha S.R., Chung S., Reader B.F., Yenigalla A., Zhang L., Shapiro J.P., Wisler J., Christman J.W., Maddipati K. (2022). Lipidomic Profiling of Bronchoalveolar Lavage Fluid Extracellular Vesicles Indicates Their Involvement in Lipopolysaccharide-Induced Acute Lung Injury. J. Innate Immun..

[119] Shaba E., Landi C., Carleo A., Vantaggiato L., Paccagnini E., Gentile M., Bianchi L., Lupetti P., Bargagli E., Prasse A. (2021). Proteome Characterization of BALF Extracellular Vesicles in Idiopathic Pulmonary Fibrosis: Unveiling Undercover Molecular Pathways. Int. J. Mol. Sci..

[120] Turchinovich A., Drapkina O., Tonevitsky A. (2019). Transcriptome of Extracellular Vesicles: State-of-the-Art. Front. Immunol..

[121] Kaur G., Maremanda K.P., Campos M., Chand H.S., Li F., Hirani N., Haseeb M.A., Li D., Rahman I. (2021). Distinct Exosomal miRNA Profiles from BALF and Lung Tissue of COPD and IPF Patients. Int. J. Mol. Sci..

[122] Lee S.E., Park H.Y., Hur J.Y., Kim H.J., Kim I.A., Kim W.S., Lee K.Y. (2021). Genomic profiling of extracellular vesicle-derived DNA from bronchoalveolar lavage fluid of patients with lung adenocarcinoma. Transl. Lung Cancer Res..

[123] Zaborowski M.P., Balaj L., Breakefield X.O., Lai C.P. (2015). Extracellular Vesicles: Composition, Biological Relevance, and Methods of Study. Bioscience.

[124] Kruh-Garcia N.A., Schorey J.S., Dobos K.M., Cardona P. (2012). Exosomes: New Tuberculosis Biomarkers—Prospects From the Bench to the Clinic. Understanding Tuberculosis—Global Experiences and Innovative Approaches to the Diagnosis.

[125] Sun T., Kalionis B., Lv G., Xia S., Gao W. (2015). Role of Exosomal Noncoding RNAs in Lung Carcinogenesis. Biomed Res. Int..

[126] Verderio C., Muzio L., Turola E., Bergami A., Novellino L., Ruffini F., Riganti L., Corradini I., Francolini M., Garzetti L. (2012). Myeloid microvesicles are a marker and therapeutic target for neuroinflammation. Ann. Neurol..

[127] Hood J.L. (2016). Post isolation modification of exosomes for nanomedicine applications. Nanomedicine.

[128] Caby M.P., Lankar D., Vincendeau-Scherrer C., Raposo G., Bonnerot C. (2005). Exosomal-like vesicles are present in human blood plasma. Int. Immunol..

[129] Sanchez-Vidaurre S., Eldh M., Larssen P., Daham K., Martinez-Bravo M.J., Dahlen S.E., Dahlen B., van Hage M., Gabrielsson S. (2017). RNA-containing exosomes in induced sputum of asthmatic patients. J. Allergy Clin. Immunol..

[130] Yagi Y., Ohkubo T., Kawaji H., Machida A., Miyata H., Goda S., Roy S., Hayashizaki Y., Suzuki H., Yokota T. (2017). Next-generation sequencing-based small RNA profiling of cerebrospinal fluid exosomes. Neurosci. Lett..

[131] Admyre C., Grunewald J., Thyberg J., Gripenback S., Tornling G., Eklund A., Scheynius A., Gabrielsson S. (2003). Exosomes with major histocompatibility complex class II and co-stimulatory molecules are present in human BAL fluid. Eur. Respir. J..

[132] Street J.M., Koritzinsky E.H., Glispie D.M., Star R.A., Yuen P.S. (2017). Urine Exosomes: An Emerging Trove of Biomarkers. Adv. Clin. Chem..

[133] Kulshreshtha A., Ahmad T., Agrawal A., Ghosh B. (2013). Proinflammatory role of epithelial cell-derived exosomes in allergic airway inflammation. J. Allergy Clin. Immunol..

[134] Cheng L., Zhang K., Wu S., Cui M., Xu T. (2017). Focus on Mesenchymal Stem Cell-Derived Exosomes: Opportunities and Challenges in Cell-Free Therapy. Stem Cells Int..

[135] Liu K., Luo X., Lv Z.Y., Zhang Y.J., Meng Z., Li J., Meng C.X., Qiang H.F., Hou C.Y., Hou L. (2021). Macrophage-Derived Exosomes Promote Bone Mesenchymal Stem Cells Towards Osteoblastic Fate Through microRNA-21a-5p. Front. Bioeng. Biotechnol..

[136] Pitt J.M., Charrier M., Viaud S., Andre F., Besse B., Chaput N., Zitvogel L. (2014). Dendritic cell-derived exosomes as immunotherapies in the fight against cancer. J. Immunol..

[137] Li D., Wang Y., Jin X., Hu D., Xia C., Xu H., Hu J. (2020). NK cell-derived exosomes carry miR-207 and alleviate depression-like symptoms in mice. J. Neuroinflamm..

[138] Cai Z., Yang F., Yu L., Yu Z., Jiang L., Wang Q., Yang Y., Wang L., Cao X., Wang J. (2012). Activated T cell exosomes promote tumor invasion via Fas signaling pathway. J. Immunol..

[139] Admyre C., Bohle B., Johansson S.M., Focke-Tejkl M., Valenta R., Scheynius A., Gabrielsson S. (2007). B cell-derived exosomes can present allergen peptides and activate allergen-specific T cells to proliferate and produce TH2-like cytokines. J. Allergy Clin. Immunol..

[140] Morishita M., Takahashi Y., Nishikawa M., Takakura Y. (2017). Pharmacokinetics of Exosomes-An Important Factor for Elucidating the Biological Roles of Exosomes and for the Development of Exosome-Based Therapeutics. J. Pharm. Sci..

[141] Munagala R., Aqil F., Jeyabalan J., Gupta R.C. (2016). Bovine milk-derived exosomes for drug delivery. Cancer Lett..

[142] Gangadaran P., Li X.J., Lee H.W., Oh J.M., Kalimuthu S., Rajendran R.L., Son S.H., Baek S.H., Singh T.D., Zhu L. (2017). A new bioluminescent reporter system to study the biodistribution of systematically injected tumor-derived bioluminescent extracellular vesicles in mice. Oncotarget.

[143] Conlan R.S., Pisano S., Oliveira M.I., Ferrari M., Mendes Pinto I. (2017). Exosomes as Reconfigurable Therapeutic Systems. Trends Mol. Med..

[144] Vlassov A.V., Magdaleno S., Setterquist R., Conrad R. (2012). Exosomes: Current knowledge of their composition, biological functions, and diagnostic and therapeutic potentials. Biochim. Biophys. Acta.

[145] Mathivanan S., Fahner C.J., Reid G.E., Simpson R.J. (2012). ExoCarta 2012: Database of exosomal proteins, RNA and lipids. Nucleic Acids Res..

[146] Kowal J., Tkach M., Thery C. (2014). Biogenesis and secretion of exosomes. Curr. Opin. Cell Biol..

[147] Tickner J.A., Urquhart A.J., Stephenson S.A., Richard D.J., O’Byrne K.J. (2014). Functions and therapeutic roles of exosomes in cancer. Front. Oncol..

[148] Segura E., Amigorena S., Thery C. (2005). Mature dendritic cells secrete exosomes with strong ability to induce antigen-specific effector immune responses. Blood Cells Mol. Dis..

[149] Kim D.K., Kang B., Kim O.Y., Choi D.S., Lee J., Kim S.R., Go G., Yoon Y.J., Kim J.H., Jang S.C. (2013). EVpedia: An integrated database of high-throughput data for systemic analyses of extracellular vesicles. J. Extracell. Vesicles.

[150] Kosaka N., Iguchi H., Yoshioka Y., Takeshita F., Matsuki Y., Ochiya T. (2010). Secretory mechanisms and intercellular transfer of microRNAs in living cells. J. Biol. Chem..

[151] Bobrie A., Colombo M., Raposo G., Thery C. (2011). Exosome secretion: Molecular mechanisms and roles in immune responses. Traffic.

[152] Anand S., Samuel M., Kumar S., Mathivanan S. (2019). Ticket to a bubble ride: Cargo sorting into exosomes and extracellular vesicles. Biochim. Biophys. Acta Proteins Proteom..

[153] Elsharkasy O.M., Nordin J.Z., Hagey D.W., de Jong O.G., Schiffelers R.M., Andaloussi S.E., Vader P. (2020). Extracellular vesicles as drug delivery systems: Why and how?. Adv. Drug Deliv. Rev..

[154] Turchinovich A., Samatov T.R., Tonevitsky A.G., Burwinkel B. (2013). Circulating miRNAs: Cell-cell communication function?. Front. Genet..

[155] Mittelbrunn M., Gutierrez-Vazquez C., Villarroya-Beltri C., Gonzalez S., Sanchez-Cabo F., Gonzalez M.A., Bernad A., Sanchez-Madrid F. (2011). Unidirectional transfer of microRNA-loaded exosomes from T cells to antigen-presenting cells. Nat. Commun..

[156] Valadi H., Ekstrom K., Bossios A., Sjostrand M., Lee J.J., Lotvall J.O. (2007). Exosome-mediated transfer of mRNAs and microRNAs is a novel mechanism of genetic exchange between cells. Nat. Cell Biol..

[157] Nazimek K., Bryniarski K., Santocki M., Ptak W. (2015). Exosomes as mediators of intercellular communication: Clinical implications. Pol. Arch. Med. Wewn..

[158] Wang M., Yu F., Ding H., Wang Y., Li P., Wang K. (2019). Emerging Function and Clinical Values of Exosomal MicroRNAs in Cancer. Mol. Ther. Nucleic Acids.

[159] Record M., Carayon K., Poirot M., Silvente-Poirot S. (2014). Exosomes as new vesicular lipid transporters involved in cell-cell communication and various pathophysiologies. Biochim. Biophys. Acta.

[160] Schorey J.S., Bhatnagar S. (2008). Exosome function: From tumor immunology to pathogen biology. Traffic.

[161] Keerthikumar S., Gangoda L., Liem M., Fonseka P., Atukorala I., Ozcitti C., Mechler A., Adda C.G., Ang C.S., Mathivanan S. (2015). Proteogenomic analysis reveals exosomes are more oncogenic than ectosomes. Oncotarget.

[162] Li C.J., Liu Y., Chen Y., Yu D., Williams K.J., Liu M.L. (2013). Novel proteolytic microvesicles released from human macrophages after exposure to tobacco smoke. Am. J. Pathol..

[163] Bernimoulin M., Waters E.K., Foy M., Steele B.M., Sullivan M., Falet H., Walsh M.T., Barteneva N., Geng J.G., Hartwig J.H. (2009). Differential stimulation of monocytic cells results in distinct populations of microparticles. J. Thromb. Haemost..

[164] Samii A., Razmkhah F. (2020). Transformation of Hematopoietic Stem and Progenitor Cells by Leukemia Extracellular Vesicles: A Step Toward Leukemogenesis. Stem Cell Rev. Rep..

[165] Cavallero S., Riccobono D., Drouet M., Francois S. (2020). MSC-Derived Extracellular Vesicles: New Emergency Treatment to Limit the Development of Radiation-Induced Hematopoietic Syndrome?. Health Phys..

[166] Roberts C.T., Kurre P. (2013). Vesicle trafficking and RNA transfer add complexity and connectivity to cell-cell communication. Cancer Res..

[167] Weerheim A.M., Kolb A.M., Sturk A., Nieuwland R. (2002). Phospholipid composition of cell-derived microparticles determined by one-dimensional high-performance thin-layer chromatography. Anal. Biochem..

[168] Thery C., Ostrowski M., Segura E. (2009). Membrane vesicles as conveyors of immune responses. Nat. Rev. Immunol..

[169] Gasser O., Hess C., Miot S., Deon C., Sanchez J.C., Schifferli J.A. (2003). Characterisation and properties of ectosomes released by human polymorphonuclear neutrophils. Exp. Cell Res..

[170] Lleo A., Zhang W., McDonald W.H., Seeley E.H., Leung P.S., Coppel R.L., Ansari A.A., Adams D.H., Afford S., Invernizzi P. (2014). Shotgun proteomics: Identification of unique protein profiles of apoptotic bodies from biliary epithelial cells. Hepatology.

[171] Mallat Z., Hugel B., Ohan J., Leseche G., Freyssinet J.M., Tedgui A. (1999). Shed membrane microparticles with procoagulant potential in human atherosclerotic plaques: A role for apoptosis in plaque thrombogenicity. Circulation.

[172] Zernecke A., Bidzhekov K., Noels H., Shagdarsuren E., Gan L., Denecke B., Hristov M., Koppel T., Jahantigh M.N., Lutgens E. (2009). Delivery of microRNA-126 by apoptotic bodies induces CXCL12-dependent vascular protection. Sci. Signal..

[173] Chiang C.Y., Chen C. (2019). Toward characterizing extracellular vesicles at a single-particle level. J. Biomed. Sci..

[174] Rock J.R., Hogan B.L. (2011). Epithelial progenitor cells in lung development, maintenance, repair, and disease. Annu. Rev. Cell Dev. Biol..

[175] Peng T., Frank D.B., Kadzik R.S., Morley M.P., Rathi K.S., Wang T., Zhou S., Cheng L., Lu M.M., Morrisey E.E. (2015). Hedgehog actively maintains adult lung quiescence and regulates repair and regeneration. Nature.

[176] Shivaraju M., Chitta U.K., Grange R.M.H., Jain I.H., Capen D., Liao L., Xu J., Ichinose F., Zapol W.M., Mootha V.K. (2021). Airway stem cells sense hypoxia and differentiate into protective solitary neuroendocrine cells. Science.

[177] Rock J.R., Onaitis M.W., Rawlins E.L., Lu Y., Clark C.P., Xue Y., Randell S.H., Hogan B.L. (2009). Basal cells as stem cells of the mouse trachea and human airway epithelium. Proc. Natl. Acad. Sci. USA.

[178] Rock J.R., Gao X., Xue Y., Randell S.H., Kong Y.Y., Hogan B.L. (2011). Notch-dependent differentiation of adult airway basal stem cells. Cell Stem Cell.

[179] Zepp J.A., Morrisey E.E. (2019). Cellular crosstalk in the development and regeneration of the respiratory system. Nat. Rev. Mol. Cell Biol..

[180] Basil M.C., Katzen J., Engler A.E., Guo M., Herriges M.J., Kathiriya J.J., Windmueller R., Ysasi A.B., Zacharias W.J., Chapman H.A. (2020). The Cellular and Physiological Basis for Lung Repair and Regeneration: Past, Present, and Future. Cell Stem Cell.

[181] Leach J.P., Morrisey E.E. (2018). Repairing the lungs one breath at a time: How dedicated or facultative are you?. Genes Dev..

[182] Kadota T., Fujita Y., Araya J., Ochiya T., Kuwano K. (2022). Extracellular vesicle-mediated cellular crosstalk in lung repair, remodelling and regeneration. Eur. Respir. Rev..

[183] Letsiou E., Bauer N. (2018). Endothelial Extracellular Vesicles in Pulmonary Function and Disease. Curr. Top. Membr..

[184] Fujita Y., Kadota T., Araya J., Ochiya T., Kuwano K. (2018). Extracellular Vesicles: New Players in Lung Immunity. Am. J. Respir. Cell Mol. Biol..

[185] Haggadone M.D., Peters-Golden M. (2018). Microenvironmental Influences on Extracellular Vesicle-Mediated Communication in the Lung. Trends Mol. Med..

[186] Su G., Ma X., Wei H. (2020). Multiple Biological Roles of Extracellular Vesicles in Lung Injury and Inflammation Microenvironment. Biomed Res. Int..

[187] Kadota T., Fujita Y., Araya J., Watanabe N., Fujimoto S., Kawamoto H., Minagawa S., Hara H., Ohtsuka T., Yamamoto Y. (2021). Human bronchial epithelial cell-derived extracellular vesicle therapy for pulmonary fibrosis via inhibition of TGF-beta-WNT crosstalk. J. Extracell. Vesicles.

[188] Quan Y., Wang Z., Gong L., Peng X., Richard M.A., Zhang J., Fornage M., Alcorn J.L., Wang D. (2017). Exosome miR-371b-5p promotes proliferation of lung alveolar progenitor type II cells by using PTEN to orchestrate the PI3K/Akt signaling. Stem Cell Res. Ther..

[189] Mitchell A., Wanczyk H., Jensen T., Finck C. (2020). Human induced pluripotent stem cells ameliorate hyperoxia-induced lung injury in a mouse model. Am. J. Transl. Res..

[190] Haj-Salem I., Plante S., Gounni A.S., Rouabhia M., Chakir J. (2018). Fibroblast-derived exosomes promote epithelial cell proliferation through TGF-beta2 signalling pathway in severe asthma. Allergy.

[191] Kadota T., Yoshioka Y., Fujita Y., Araya J., Minagawa S., Hara H., Miyamoto A., Suzuki S., Fujimori S., Kohno T. (2020). Extracellular Vesicles from Fibroblasts Induce Epithelial-Cell Senescence in Pulmonary Fibrosis. Am. J. Respir. Cell Mol. Biol..

[192] Yoshimura A., Naka T., Kubo M. (2007). SOCS proteins, cytokine signalling and immune regulation. Nat. Rev. Immunol..

[193] Draijer C., Speth J.M., Penke L.R.K., Zaslona Z., Bazzill J.D., Lugogo N., Huang Y.J., Moon J.J., Peters-Golden M. (2020). Resident alveolar macrophage-derived vesicular SOCS3 dampens allergic airway inflammation. FASEB J..

[194] Zhu Z., Zhang D., Lee H., Menon A.A., Wu J., Hu K., Jin Y. (2017). Macrophage-derived apoptotic bodies promote the proliferation of the recipient cells via shuttling microRNA-221/222. J. Leukoc. Biol..

[195] Guiot J., Cambier M., Boeckx A., Henket M., Nivelles O., Gester F., Louis E., Malaise M., Dequiedt F., Louis R. (2020). Macrophage-derived exosomes attenuate fibrosis in airway epithelial cells through delivery of antifibrotic miR-142-3p. Thorax.

[196] Khatri M., Richardson L.A., Meulia T. (2018). Mesenchymal stem cell-derived extracellular vesicles attenuate influenza virus-induced acute lung injury in a pig model. Stem Cell Res. Ther..

[197] Tang X.D., Shi L., Monsel A., Li X.Y., Zhu H.L., Zhu Y.G., Qu J.M. (2017). Mesenchymal Stem Cell Microvesicles Attenuate Acute Lung Injury in Mice Partly Mediated by Ang-1 mRNA. Stem Cells.

[198] Monsel A., Zhu Y.G., Gennai S., Hao Q., Hu S., Rouby J.J., Rosenzwajg M., Matthay M.A., Lee J.W. (2015). Therapeutic Effects of Human Mesenchymal Stem Cell-derived Microvesicles in Severe Pneumonia in Mice. Am. J. Respir. Crit. Care Med..

[199] Zhu Y.G., Feng X.M., Abbott J., Fang X.H., Hao Q., Monsel A., Qu J.M., Matthay M.A., Lee J.W. (2014). Human mesenchymal stem cell microvesicles for treatment of Escherichia coli endotoxin-induced acute lung injury in mice. Stem Cells.

[200] Dutra Silva J., Su Y., Calfee C.S., Delucchi K.L., Weiss D., McAuley D.F., O’Kane C., Krasnodembskaya A.D. (2021). Mesenchymal stromal cell extracellular vesicles rescue mitochondrial dysfunction and improve barrier integrity in clinically relevant models of ARDS. Eur. Respir. J..

[201] Yi X., Wei X., Lv H., An Y., Li L., Lu P., Yang Y., Zhang Q., Yi H., Chen G. (2019). Exosomes derived from microRNA-30b-3p-overexpressing mesenchymal stem cells protect against lipopolysaccharide-induced acute lung injury by inhibiting SAA3. Exp. Cell Res..

[202] Li J.W., Wei L., Han Z., Chen Z. (2019). Mesenchymal stromal cells-derived exosomes alleviate ischemia/reperfusion injury in mouse lung by transporting anti-apoptotic miR-21-5p. Eur. J. Pharmacol..

[203] Shin J.Y., Park H.J., Kim H.N., Oh S.H., Bae J.S., Ha H.J., Lee P.H. (2014). Mesenchymal stem cells enhance autophagy and increase beta-amyloid clearance in Alzheimer disease models. Autophagy.

[204] Dang S., Xu H., Xu C., Cai W., Li Q., Cheng Y., Jin M., Wang R.X., Peng Y., Zhang Y. (2014). Autophagy regulates the therapeutic potential of mesenchymal stem cells in experimental autoimmune encephalomyelitis. Autophagy.

[205] Wei J., Song Y., Du Z., Yu F., Zhang Y., Jiang N., Ge X. (2020). Exosomes derived from human exfoliated deciduous teeth ameliorate adult bone loss in mice through promoting osteogenesis. J. Mol. Histol..

[206] Nolte-’t Hoen E.N., Buschow S.I., Anderton S.M., Stoorvogel W., Wauben M.H. (2009). Activated T cells recruit exosomes secreted by dendritic cells via LFA-1. Blood.

[207] Zhang B., Wang M., Gong A., Zhang X., Wu X., Zhu Y., Shi H., Wu L., Zhu W., Qian H. (2015). HucMSC-Exosome Mediated-Wnt4 Signaling Is Required for Cutaneous Wound Healing. Stem Cells.

[208] Cui X., He Z., Liang Z., Chen Z., Wang H., Zhang J. (2017). Exosomes From Adipose-derived Mesenchymal Stem Cells Protect the Myocardium Against Ischemia/Reperfusion Injury Through Wnt/beta-Catenin Signaling Pathway. J. Cardiovasc. Pharmacol..

[209] Ratajczak J., Miekus K., Kucia M., Zhang J., Reca R., Dvorak P., Ratajczak M.Z. (2006). Embryonic stem cell-derived microvesicles reprogram hematopoietic progenitors: Evidence for horizontal transfer of mRNA and protein delivery. Leukemia.

[210] Bang C., Batkai S., Dangwal S., Gupta S.K., Foinquinos A., Holzmann A., Just A., Remke J., Zimmer K., Zeug A. (2014). Cardiac fibroblast-derived microRNA passenger strand-enriched exosomes mediate cardiomyocyte hypertrophy. J. Clin. Investig..

[211] Zamani P., Fereydouni N., Butler A.E., Navashenaq J.G., Sahebkar A. (2019). The therapeutic and diagnostic role of exosomes in cardiovascular diseases. Trends Cardiovasc. Med..

[212] Howitt J., Hill A.F. (2016). Exosomes in the Pathology of Neurodegenerative Diseases. J. Biol. Chem..

[213] Allegretta C., D’Amico E., Manuti V., Avolio C., Conese M. (2022). Mesenchymal Stem Cell-Derived Extracellular Vesicles and Their Therapeutic Use in Central Nervous System Demyelinating Disorders. Int. J. Mol. Sci..

[214] Osaki M., Okada F. (2019). Exosomes and Their Role in Cancer Progression. Yonago Acta Med..

[215] Chan B.D., Wong W.Y., Lee M.M., Cho W.C., Yee B.K., Kwan Y.W., Tai W.C. (2019). Exosomes in Inflammation and Inflammatory Disease. Proteomics.

[216] Mathivanan S., Ji H., Simpson R.J. (2010). Exosomes: Extracellular organelles important in intercellular communication. J. Proteom..

[217] Mulcahy L.A., Pink R.C., Carter D.R. (2014). Routes and mechanisms of extracellular vesicle uptake. J. Extracell. Vesicles.

[218] Yoon J.H., Ashktorab H., Smoot D.T., Nam S.W., Hur H., Park W.S. (2020). Uptake and tumor-suppressive pathways of exosome-associated GKN1 protein in gastric epithelial cells. Gastric Cancer.

[219] Feng D., Zhao W.L., Ye Y.Y., Bai X.C., Liu R.Q., Chang L.F., Zhou Q., Sui S.F. (2010). Cellular internalization of exosomes occurs through phagocytosis. Traffic.

[220] Eguchi S., Takefuji M., Sakaguchi T., Ishihama S., Mori Y., Tsuda T., Takikawa T., Yoshida T., Ohashi K., Shimizu Y. (2019). Cardiomyocytes capture stem cell-derived, anti-apoptotic microRNA-214 via clathrin-mediated endocytosis in acute myocardial infarction. J. Biol. Chem..

[221] Kadota T., Fujita Y., Yoshioka Y., Araya J., Kuwano K., Ochiya T. (2016). Extracellular Vesicles in Chronic Obstructive Pulmonary Disease. Int. J. Mol. Sci..

[222] Berumen Sanchez G., Bunn K.E., Pua H.H., Rafat M. (2021). Extracellular vesicles: Mediators of intercellular communication in tissue injury and disease. Cell Commun. Signal..

[223] Kesimer M., Scull M., Brighton B., DeMaria G., Burns K., O’Neal W., Pickles R.J., Sheehan J.K. (2009). Characterization of exosome-like vesicles released from human tracheobronchial ciliated epithelium: A possible role in innate defense. FASEB J..

[224] Purghe B., Manfredi M., Ragnoli B., Baldanzi G., Malerba M. (2021). Exosomes in chronic respiratory diseases. Biomed. Pharm..

[225] Alipoor S.D., Adcock I.M., Garssen J., Mortaz E., Varahram M., Mirsaeidi M., Velayati A. (2016). The roles of miRNAs as potential biomarkers in lung diseases. Eur. J. Pharmacol..

[226] Hough K.P., Chanda D., Duncan S.R., Thannickal V.J., Deshane J.S. (2017). Exosomes in immunoregulation of chronic lung diseases. Allergy.

[227] Hattrup C.L., Gendler S.J. (2008). Structure and function of the cell surface (tethered) mucins. Annu. Rev. Physiol..

[228] Fujita Y., Araya J., Ito S., Kobayashi K., Kosaka N., Yoshioka Y., Kadota T., Hara H., Kuwano K., Ochiya T. (2015). Suppression of autophagy by extracellular vesicles promotes myofibroblast differentiation in COPD pathogenesis. J. Extracell. Vesicles.

[229] Xu H., Ling M., Xue J., Dai X., Sun Q., Chen C., Liu Y., Zhou L., Liu J., Luo F. (2018). Exosomal microRNA-21 derived from bronchial epithelial cells is involved in aberrant epithelium-fibroblast cross-talk in COPD induced by cigarette smoking. Theranostics.

[230] Moon H.G., Kim S.H., Gao J., Quan T., Qin Z., Osorio J.C., Rosas I.O., Wu M., Tesfaigzi Y., Jin Y. (2014). CCN1 secretion and cleavage regulate the lung epithelial cell functions after cigarette smoke. Am. J. Physiol. Lung Cell Mol. Physiol..

[231] Yuan Z., Petree J.R., Lee F.E., Fan X., Salaita K., Guidot D.M., Sadikot R.T. (2019). Macrophages exposed to HIV viral protein disrupt lung epithelial cell integrity and mitochondrial bioenergetics via exosomal microRNA shuttling. Cell Death Dis..

[232] Ismail N., Wang Y., Dakhlallah D., Moldovan L., Agarwal K., Batte K., Shah P., Wisler J., Eubank T.D., Tridandapani S. (2013). Macrophage microvesicles induce macrophage differentiation and miR-223 transfer. Blood.

[233] Yanez-Mo M., Siljander P.R., Andreu Z., Zavec A.B., Borras F.E., Buzas E.I., Buzas K., Casal E., Cappello F., Carvalho J. (2015). Biological properties of extracellular vesicles and their physiological functions. J. Extracell. Vesicles.

[234] Qu Y., Ramachandra L., Mohr S., Franchi L., Harding C.V., Nunez G., Dubyak G.R. (2009). P2X7 receptor-stimulated secretion of MHC class II-containing exosomes requires the ASC/NLRP3 inflammasome but is independent of caspase-1. J. Immunol..

[235] Cordazzo C., Petrini S., Neri T., Lombardi S., Carmazzi Y., Pedrinelli R., Paggiaro P., Celi A. (2014). Rapid shedding of proinflammatory microparticles by human mononuclear cells exposed to cigarette smoke is dependent on Ca2+ mobilization. Inflamm. Res..

[236] De Toro J., Herschlik L., Waldner C., Mongini C. (2015). Emerging roles of exosomes in normal and pathological conditions: New insights for diagnosis and therapeutic applications. Front. Immunol..

[237] Chironi G.N., Boulanger C.M., Simon A., Dignat-George F., Freyssinet J.M., Tedgui A. (2009). Endothelial microparticles in diseases. Cell Tissue Res..

[238] Heiss C., Amabile N., Lee A.C., Real W.M., Schick S.F., Lao D., Wong M.L., Jahn S., Angeli F.S., Minasi P. (2008). Brief secondhand smoke exposure depresses endothelial progenitor cells activity and endothelial function: Sustained vascular injury and blunted nitric oxide production. J. Am. Coll. Cardiol..

[239] Lockett A.D., Brown M.B., Santos-Falcon N., Rush N.I., Oueini H., Oberle A.J., Bolanis E., Fragoso M.A., Petrusca D.N., Serban K.A. (2014). Active trafficking of alpha 1 antitrypsin across the lung endothelium. PLoS ONE.

[240] Strulovici-Barel Y., Staudt M.R., Krause A., Gordon C., Tilley A.E., Harvey B.G., Kaner R.J., Hollmann C., Mezey J.G., Bitter H. (2016). Persistence of circulating endothelial microparticles in COPD despite smoking cessation. Thorax.

[241] Barak O.F., Mladinov S., Hoiland R.L., Tremblay J.C., Thom S.R., Yang M., Mijacika T., Dujic Z. (2017). Disturbed blood flow worsens endothelial dysfunction in moderate-severe chronic obstructive pulmonary disease. Sci. Rep..

[242] Nieri D., Daniele M., Lombardi S., Bazzan E., Santerini S., De Cusatis G., Vagaggini B., Cosio M.G., Saetta M., Paggiaro P. (2021). Circulating Extracellular Vesicles Are Associated with Disease Severity and Interleukin-6 Levels in COPD: A Pilot Study. J. Clin. Med..

[243] Lascano J., Katz J., Cearras M., Campos M. (2021). Association of Systemic Endothelial-Derived and Platelet-Derived Microparticles With Clinical Outcomes in Chronic Obstructive Pulmonary Disease. Chronic. Obstr. Pulm. Dis..

[244] Liu H., Ding L., Zhang Y., Ni S. (2014). Circulating endothelial microparticles involved in lung function decline in a rat exposed in cigarette smoke maybe from apoptotic pulmonary capillary endothelial cells. J. Thorac. Dis..

[245] Useckaite Z., Ward M.P., Trappe A., Reilly R., Lennon J., Davage H., Matallanas D., Cassidy H., Dillon E.T., Brennan K. (2020). Increased extracellular vesicles mediate inflammatory signalling in cystic fibrosis. Thorax.

[246] Cook-Mills J.M., Marchese M.E., Abdala-Valencia H. (2011). Vascular cell adhesion molecule-1 expression and signaling during disease: Regulation by reactive oxygen species and antioxidants. Antioxid. Redox. Signal..

[247] Donato R., Cannon B.R., Sorci G., Riuzzi F., Hsu K., Weber D.J., Geczy C.L. (2013). Functions of S100 proteins. Curr. Mol. Med..

[248] Porro C., Lepore S., Trotta T., Castellani S., Ratclif L., Battaglino A., Di Gioia S., Martinez M.C., Conese M., Maffione A.B. (2010). Isolation and characterization of microparticles in sputum of cystic fibrosis patients. Respir. Res..

[249] Porro C., Di Gioia S., Trotta T., Lepore S., Panaro M.A., Battaglino A., Ratclif L., Castellani S., Bufo P., Martinez M.C. (2013). Pro-inflammatory effect of cystic fibrosis sputum microparticles in the murine lung. J. Cyst. Fibros..

[250] Rollet-Cohen V., Bourderioux M., Lipecka J., Chhuon C., Jung V.A., Mesbahi M., Nguyen-Khoa T., Guerin-Pfyffer S., Schmitt A., Edelman A. (2018). Comparative proteomics of respiratory exosomes in cystic fibrosis, primary ciliary dyskinesia and asthma. J. Proteom..

[251] Martinez-Aleman S.R., Campos-Garcia L., Palma-Nicolas J.P., Hernandez-Bello R., Gonzalez G.M., Sanchez-Gonzalez A. (2017). Understanding the Entanglement: Neutrophil Extracellular Traps (NETs) in Cystic Fibrosis. Front. Cell Infect. Microbiol..

[252] Schroll A., Eller K., Feistritzer C., Nairz M., Sonnweber T., Moser P.A., Rosenkranz A.R., Theurl I., Weiss G. (2012). Lipocalin-2 ameliorates granulocyte functionality. Eur. J. Immunol..

[253] Lorenz E., Muhlebach M.S., Tessier P.A., Alexis N.E., Duncan Hite R., Seeds M.C., Peden D.B., Meredith W. (2008). Different expression ratio of S100A8/A9 and S100A12 in acute and chronic lung diseases. Respir. Med..

[254] Rouleau P., Vandal K., Ryckman C., Poubelle P.E., Boivin A., Talbot M., Tessier P.A. (2003). The calcium-binding protein S100A12 induces neutrophil adhesion, migration, and release from bone marrow in mouse at concentrations similar to those found in human inflammatory arthritis. Clin. Immunol..

[255] Foell D., Seeliger S., Vogl T., Koch H.G., Maschek H., Harms E., Sorg C., Roth J. (2003). Expression of S100A12 (EN-RAGE) in cystic fibrosis. Thorax.

[256] Forrest O.A., Dobosh B., Ingersoll S.A., Rao S., Rojas A., Laval J., Alvarez J.A., Brown M.R., Tangpricha V., Tirouvanziam R. (2022). Neutrophil-derived extracellular vesicles promote feed-forward inflammasome signaling in cystic fibrosis airways. J. Leukoc. Biol..

[257] Xie N., Tan Z., Banerjee S., Cui H., Ge J., Liu R.M., Bernard K., Thannickal V.J., Liu G. (2015). Glycolytic Reprogramming in Myofibroblast Differentiation and Lung Fibrosis. Am. J. Respir. Crit. Care Med..

[258] Spanjer A.I., Baarsma H.A., Oostenbrink L.M., Jansen S.R., Kuipers C.C., Lindner M., Postma D.S., Meurs H., Heijink I.H., Gosens R. (2016). TGF-beta-induced profibrotic signaling is regulated in part by the WNT receptor Frizzled-8. FASEB J..

[259] Wang Z., Li R., Zhong R. (2018). Extracellular matrix promotes proliferation, migration and adhesion of airway smooth muscle cells in a rat model of chronic obstructive pulmonary disease via upregulation of the PI3K/AKT signaling pathway. Mol. Med. Rep..

[260] Moon H.G., Zheng Y., An C.H., Kim Y.K., Jin Y. (2013). CCN1 secretion induced by cigarette smoking extracts augments IL-8 release from bronchial epithelial cells. PLoS ONE.

[261] Foronjy R.F., Okada Y., Cole R., D’Armiento J. (2003). Progressive adult-onset emphysema in transgenic mice expressing human MMP-1 in the lung. Am. J. Physiol. Lung Cell Mol. Physiol..

[262] Geraghty P., Dabo A.J., D’Armiento J. (2011). TLR4 protein contributes to cigarette smoke-induced matrix metalloproteinase-1 (MMP-1) expression in chronic obstructive pulmonary disease. J. Biol. Chem..

[263] Koyama S., Sato E., Haniuda M., Numanami H., Nagai S., Izumi T. (2002). Decreased level of vascular endothelial growth factor in bronchoalveolar lavage fluid of normal smokers and patients with pulmonary fibrosis. Am. J. Respir. Crit. Care Med..

[264] Tetley T.D. (2002). Macrophages and the pathogenesis of COPD. Chest.

[265] Hiemstra P.S. (2013). Altered macrophage function in chronic obstructive pulmonary disease. Ann. Am. Thorac. Soc..

[266] Hautamaki R.D., Kobayashi D.K., Senior R.M., Shapiro S.D. (1997). Requirement for macrophage elastase for cigarette smoke-induced emphysema in mice. Science.

[267] Molet S., Belleguic C., Lena H., Germain N., Bertrand C.P., Shapiro S.D., Planquois J.M., Delaval P., Lagente V. (2005). Increase in macrophage elastase (MMP-12) in lungs from patients with chronic obstructive pulmonary disease. Inflamm. Res..

[268] Lacedonia D., Carpagnano G.E., Trotta T., Palladino G.P., Panaro M.A., Zoppo L.D., Foschino Barbaro M.P., Porro C. (2016). Microparticles in sputum of COPD patients: A potential biomarker of the disease?. Int. J. Chron. Obstruct. Pulmon. Dis..

[269] Morel N., Morel O., Petit L., Hugel B., Cochard J.F., Freyssinet J.M., Sztark F., Dabadie P. (2008). Generation of procoagulant microparticles in cerebrospinal fluid and peripheral blood after traumatic brain injury. J. Trauma.

[270] Genschmer K.R., Russell D.W., Lal C., Szul T., Bratcher P.E., Noerager B.D., Abdul Roda M., Xu X., Rezonzew G., Viera L. (2019). Activated PMN Exosomes: Pathogenic Entities Causing Matrix Destruction and Disease in the Lung. Cell.

[271] Margaroli C., Madison M.C., Viera L., Russell D.W., Gaggar A., Genschmer K.R., Blalock J.E. (2022). An in vivo model for extracellular vesicle-induced emphysema. JCI Insight.

[272] O’Reilly P., Jackson P.L., Noerager B., Parker S., Dransfield M., Gaggar A., Blalock J.E. (2009). N-alpha-PGP and PGP, potential biomarkers and therapeutic targets for COPD. Respir. Res..

[273] Zhang Y., Hu Y.W., Zheng L., Wang Q. (2017). Characteristics and Roles of Exosomes in Cardiovascular Disease. DNA Cell Biol..

[274] Jansen F., Li Q. (2017). Exosomes as Diagnostic Biomarkers in Cardiovascular Diseases. Adv. Exp. Med. Biol..

[275] Kanninen K.M., Bister N., Koistinaho J., Malm T. (2016). Exosomes as new diagnostic tools in CNS diseases. Biochim. Biophys. Acta.

[276] Zhang W., Zhou X., Zhang H., Yao Q., Liu Y., Dong Z. (2016). Extracellular vesicles in diagnosis and therapy of kidney diseases. Am. J. Physiol. Renal Physiol..

[277] Masyuk A.I., Masyuk T.V., Larusso N.F. (2013). Exosomes in the pathogenesis, diagnostics and therapeutics of liver diseases. J. Hepatol..

[278] Alipoor S.D., Mortaz E., Varahram M., Movassaghi M., Kraneveld A.D., Garssen J., Adcock I.M. (2018). The Potential Biomarkers and Immunological Effects of Tumor-Derived Exosomes in Lung Cancer. Front. Immunol..

[279] Fitts C.A., Ji N., Li Y., Tan C. (2019). Exploiting Exosomes in Cancer Liquid Biopsies and Drug Delivery. Adv. Healthc. Mater..

[280] Fujita Y., Yoshioka Y., Ochiya T. (2016). Extracellular vesicle transfer of cancer pathogenic components. Cancer Sci..

[281] Kosaka N., Yoshioka Y., Fujita Y., Ochiya T. (2016). Versatile roles of extracellular vesicles in cancer. J. Clin. Investig..

[282] Yoshioka Y., Kosaka N., Konishi Y., Ohta H., Okamoto H., Sonoda H., Nonaka R., Yamamoto H., Ishii H., Mori M. (2014). Ultra-sensitive liquid biopsy of circulating extracellular vesicles using ExoScreen. Nat. Commun..

[283] Clayton A., Buschmann D., Byrd J.B., Carter D.R.F., Cheng L., Compton C., Daaboul G., Devitt A., Falcon-Perez J.M., Gardiner C. (2018). Summary of the ISEV workshop on extracellular vesicles as disease biomarkers, held in Birmingham, UK, during December 2017. J. Extracell. Vesicles.

[284] De Jong O.G., Verhaar M.C., Chen Y., Vader P., Gremmels H., Posthuma G., Schiffelers R.M., Gucek M., van Balkom B.W. (2012). Cellular stress conditions are reflected in the protein and RNA content of endothelial cell-derived exosomes. J. Extracell. Vesicles.

[285] Beninson L.A., Fleshner M. (2014). Exosomes: An emerging factor in stress-induced immunomodulation. Semin. Immunol..

[286] Iraci N., Leonardi T., Gessler F., Vega B., Pluchino S. (2016). Focus on Extracellular Vesicles: Physiological Role and Signalling Properties of Extracellular Membrane Vesicles. Int. J. Mol. Sci..

[287] Russell A.E., Sneider A., Witwer K.W., Bergese P., Bhattacharyya S.N., Cocks A., Cocucci E., Erdbrugger U., Falcon-Perez J.M., Freeman D.W. (2019). Biological membranes in EV biogenesis, stability, uptake, and cargo transfer: An ISEV position paper arising from the ISEV membranes and EVs workshop. J. Extracell. Vesicles.

[288] Shoki A.H., Mayer-Hamblett N., Wilcox P.G., Sin D.D., Quon B.S. (2013). Systematic review of blood biomarkers in cystic fibrosis pulmonary exacerbations. Chest.

[289] Conese M., Tirelli A.S., Alicandro G., Di Gioia S., Carbone A., Castellani S., Colombo C. (2016). Biomarkers of Inflammation and Remodelling in Cystic Fibrosis. Clin. Immunol. Endocr. Metab. Drugs.

[290] Liou T.G., Adler F.R., Keogh R.H., Li Y., Jensen J.L., Walsh W., Packer K., Clark T., Carveth H., Chen J. (2012). Sputum biomarkers and the prediction of clinical outcomes in patients with cystic fibrosis. PLoS ONE.

[291] Jain R., Baines A., Khan U., Wagner B.D., Sagel S.D. (2021). Evaluation of airway and circulating inflammatory biomarkers for cystic fibrosis drug development. J. Cyst. Fibros..

[292] Gauthier S., Pranke I., Jung V., Martignetti L., Stoven V., Nguyen-Khoa T., Semeraro M., Hinzpeter A., Edelman A., Guerrera I.C. (2020). Urinary Exosomes of Patients with Cystic Fibrosis Unravel CFTR-Related Renal Disease. Int. J. Mol. Sci..

[293] Gaggar A., Jackson P.L., Noerager B.D., O’Reilly P.J., McQuaid D.B., Rowe S.M., Clancy J.P., Blalock J.E. (2008). A novel proteolytic cascade generates an extracellular matrix-derived chemoattractant in chronic neutrophilic inflammation. J. Immunol..

[294] Snelgrove R.J., Jackson P.L., Hardison M.T., Noerager B.D., Kinloch A., Gaggar A., Shastry S., Rowe S.M., Shim Y.M., Hussell T. (2010). A critical role for LTA4H in limiting chronic pulmonary neutrophilic inflammation. Science.

[295] Weathington N.M., van Houwelingen A.H., Noerager B.D., Jackson P.L., Kraneveld A.D., Galin F.S., Folkerts G., Nijkamp F.P., Blalock J.E. (2006). A novel peptide CXCR ligand derived from extracellular matrix degradation during airway inflammation. Nat. Med..

[296] Szul T., Bratcher P.E., Fraser K.B., Kong M., Tirouvanziam R., Ingersoll S., Sztul E., Rangarajan S., Blalock J.E., Xu X. (2016). Toll-Like Receptor 4 Engagement Mediates Prolyl Endopeptidase Release from Airway Epithelia via Exosomes. Am. J. Respir. Cell Mol. Biol..

[297] Sundar I.K., Li D., Rahman I. (2019). Proteomic Analysis of Plasma-Derived Extracellular Vesicles in Smokers and Patients with Chronic Obstructive Pulmonary Disease. ACS Omega.

[298] Gordon C., Gudi K., Krause A., Sackrowitz R., Harvey B.G., Strulovici-Barel Y., Mezey J.G., Crystal R.G. (2011). Circulating endothelial microparticles as a measure of early lung destruction in cigarette smokers. Am. J. Respir. Crit. Care Med..

[299] Takahashi T., Kobayashi S., Fujino N., Suzuki T., Ota C., He M., Yamada M., Suzuki S., Yanai M., Kurosawa S. (2012). Increased circulating endothelial microparticles in COPD patients: A potential biomarker for COPD exacerbation susceptibility. Thorax.

[300] Thomashow M.A., Shimbo D., Parikh M.A., Hoffman E.A., Vogel-Claussen J., Hueper K., Fu J., Liu C.Y., Bluemke D.A., Ventetuolo C.E. (2013). Endothelial microparticles in mild chronic obstructive pulmonary disease and emphysema. The Multi-Ethnic Study of Atherosclerosis Chronic Obstructive Pulmonary Disease study. Am. J. Respir. Crit. Care Med..

[301] Serban K.A., Rezania S., Petrusca D.N., Poirier C., Cao D., Justice M.J., Patel M., Tsvetkova I., Kamocki K., Mikosz A. (2016). Structural and functional characterization of endothelial microparticles released by cigarette smoke. Sci. Rep..

[302] Sundar I.K., Li D., Rahman I. (2019). Small RNA-sequence analysis of plasma-derived extracellular vesicle miRNAs in smokers and patients with chronic obstructive pulmonary disease as circulating biomarkers. J. Extracell. Vesicles.

[303] Tan D.B.A., Armitage J., Teo T.H., Ong N.E., Shin H., Moodley Y.P. (2017). Elevated levels of circulating exosome in COPD patients are associated with systemic inflammation. Respir. Med..

